# Neighborly social pressure and collective action: Evidence from a field experiment in Tunisia

**DOI:** 10.1371/journal.pone.0304269

**Published:** 2024-07-19

**Authors:** Prisca Jöst

**Affiliations:** 1 Cluster of Excellence “The Politics of Inequality”, University of Konstanz, Konstanz, Germany; 2 University of Marburg, Marburg, Germany; Rikkyo University, JAPAN

## Abstract

Research on political participation almost unanimously assumes that social pressure by neighbors induces collective behavior. Yet most experimental studies focus on individually based forms of political and civic behavior, such as voting and recycling, in Western industrialized societies. The paper tests the effect of neighborly social pressure on collective action in Tunisia. In a field experiment, I manipulate whether neighbors or community outsiders invite citizens to contribute to a public good (i.e., trash collection). I run the experiment in three neighborhoods of varying socioeconomic composition in Tunis (n = 1199). I do not find evidence to suggest that neighborly social pressure encourages participation in neighborhood cleanups, with low participation rates both for the neighbor and outsider contact conditions. While the effect of social pressure does not significantly vary across communities, overall participation rates do. Residents of the poor neighborhood are most likely to respond in a socially desirable way when asked about their intentions but least likely to participate. The paper also discusses some limitations of the study and outlines avenues for future research.

## Introduction

A vast literature on political participation argues that social pressure induces collective behavior, thereby overcoming social dilemmas, such as free-riding and coordination problems [1]. In particular, social pressure by neighbors and peers has been argued to effectively coordinate engagement [2–4]. Empirically, however, the link has been tested almost exclusively on less-time intense, individually-based forms of participation that do not require the coordination of the community members, such as recycling, voting, and donating money to a public good [2–9]. The well-established assumption in the literature is that social pressure is facilitated by spreading information and monitoring within local social networks [2, 3, 10]. Yet, even where the spread of information is prevented, local social ties encourage participation because entrenched social norms of engagement are activated through these networks [5]. In other words, the existence of entrenched social norms and the ability to monitor misbehavior may depend on the networks and relationship between those who monitor and those who are monitored.

This paper aims to test the conjectured argument that social pressure by neighbors increases participation in civic actions. I hypothesize that citizens will be more likely to participate in civic actions following social pressure from neighbors as compared to community outsiders (see S2 File for the pre-analysis plan and pre-registered hypotheses).

Moreover, previous work suggests that levels of local political and civic engagement should vary by neighborhood context. However, the direction of the effect is less clear. Some find that individuals in poor communities show higher levels of political and civic engagement [11, 12], partly because the poor rely on their neighbors as a safety net where state institutions are weak [13, 14]. Experimental studies in social psychology further show that the poor show more pro-social behavior and are generally more oriented toward others than the wealthy [15, 16]. Based on this previous literature, it can be hypothesized that the recruitment by neighbor mobilizers will drive higher participation in the cleanups in homogeneously poor than socioeconomically mixed and wealthy neighborhoods (pre-registered hypothesis).

However, in a recent field experiment, the authors did not find evidence that individuals of a higher socioeconomic status behave less prosocial than poorer individuals [17]. Moreover, lab experimental evidence suggests that individuals from poor neighborhoods contribute even less than citizens from wealthier neighborhoods [18]. Accordingly, one may expect poor communities to be less engaged in community activities than more affluent communities. Yet, whether this means they are also less responsive to social pressure from neighbors and peers remains an open question.

Therefore, I test the effect of social pressure on civic participation in the case of a neighborhood cleanup in Tunis and investigate how the effect varies by community social context. I acknowledge that I only focus on the main experimental hypotheses in this paper. Nonetheless, I tested all pre-registered hypotheses empirically. For a list of all pre-registered hypotheses and the empirical tests, see S6 File.

To test my expectations, I ran a field experiment (n = 1199) in which I manipulated social pressure by sending neighbors vs. community outsiders to invite citizens to participate in a neighborhood cleanup. The project mimics a design introduced by Betsy Sinclair, who sent neighbor vs. stranger canvassers to US households to measure the impact of social pressure on turnout [2]. In the following study, I send interviewers to randomly selected households to invite the heads of households to join the cleanups (see information on the sampling method File). I use face-to-face surveys and phone calls to ascertain commitment and behavior. The same design is repeated in three neighborhoods of varying socioeconomic composition.

I conducted a first research trip to Tunisia in November 2019 to prepare for this study. Regarding our clean-up sites, I identified natural areas that local stakeholders agreed to require a clean-up and to which potential participants have equal access. I also collaborated closely with the National Statistics Institute in Tunis which provided data from the 2014 census—including socioeconomic indicators on all neighborhoods in Tunis. I chose three neighborhoods from three neighboring municipalities within the Governorate of Tunis (see S5 File): Le Kram West is homogenously poor, La Marsa Corniche is homogeneously wealthy, and La Goulette Casino is a socioeconomically mixed neighborhood located at the beach which became our clean-up site. We also conducted a pilot study two weeks before we started recruiting for the first clean-up event with 64 recruited participants in Hammam-Lif (Governorate of Ben Arous). We increased the number of recruited heads of households from 300 to 400 participants in each neighborhood following low participation in the pilot study.

Civic participation in neighborhood cleanups makes a good case to study civic engagement given the higher relevance of the public good to the different social segments of society. Solid waste management is becoming an increasingly severe problem for municipalities in many countries in the Global South where population rates are rising and national economies are expanding [19, 20]. Higher spending capacities of an increasing number of citizens and continuing urbanization lead to higher waste production [21, 22]. Most municipalities collect only a small percentage of the waste; the rest end up on the street and in parks. In Tunisia’s capital city, between 0.58 and 0.82 kg of solid waste is produced per person per day (mean value of 0.65 kg) [23, 24]. The municipalities are responsible for trash collection, and citizens are asked to put their waste in plastic bags close to the nearest road where they are picked up collectively. Around 80 percent of municipal solid waste (MSW) gets collected in the big cities, yet 20 percent of MSW ends up on the streets [25]. At the same time, neighborhood cleanups are also realistic in Tunisia, and citizens organize clean-up campaigns, for example, after sit-ins and strikes [26]. During interviews in November 2019, respondents in impoverished and wealthy neighborhoods reported that they had previously heard of or personally participated in a cleanup initiative. Accordingly, neighborhood cleanups are a good case to study collective action because solid waste presents a severe problem in Tunisia, which most Tunisians are aware of [25, 26]. Thus, given the importance of the problem and the seeming awareness of it among Tunisian citizens of differing socioeconomic backgrounds, organizing a waste collection initiative should attract Tunisians from different social classes and help eliminate pre-experimental biases. This allows me to test whether individuals are more likely to attend when invited by neighbors than community outsiders in different neighborhoods.

Thus, my study moves beyond previous work that heavily draws on findings from Western, industrialized countries and tests the effect of neighborly social pressure on participation in a neighborhood cleanup in Tunisia. While I more generally expect social pressure to influence individual behavior independent of the context, differences in the extent to which individuals respond to social pressure may exist across countries. In particular, we may expect cultural and historical differences to explain variations in the extent to which people respond to social pressure from neighbors and peers. This study aims to provide first insights into the role of social pressure on participation in a context other than Europe and the US. Yet more research is needed to understand how social pressure works across countries.

## Material and methods

### Study design

I examine whether social pressure increases citizens’ participation in a field experiment conducted in Tunisia in November 2021. The following study received ethical approval from the Ethics Commission at the University of Konstanz, Germany (IRB statement 26/2021, see S7 File). Informed consent was obtained in written form from all participants. Additional information regarding the ethical, cultural, and scientific considerations specific to inclusivity in global research is included in the Supporting Information (S3 File Checklist).

As part of the experiment, I organized a cleaning campaign with my local partner organization, ELKA, at the local beaches in three neighborhoods in Tunis. Le Kram West, a homogenously poor neighborhood, La Marsa Corniche, a homogeneously wealthy neighborhood, and La Goulette Casino, a socioeconomically mixed neighborhood, are located along the coastline. The neighborhoods were selected to be able to compare contexts of different levels of socioeconomic inequality and to explore how the effect of social pressure on participation varies across contexts.

### Recruitment of participants

Trained interviewers invited 400 randomly selected heads of households in each neighborhood (one individual per household) to clean the beach. A randomly selected half of the respondents in each neighborhood are invited by neighbor recruiters, while community outsiders invited the other half (see information on the sampling in S5 File). Neighbor recruiters were interviewers who lived in the same neighborhood as the respondents. Community outsiders lived in different neighborhoods in Tunis. When visiting a household, an interviewer asked the head of the household to participate in a small (baseline) survey (see survey questions in S8 File). The survey included questions about the social demographics of the person and the household, the social ties within the neighborhood, and a series of questions that tapped into the general awareness of the problem and whether the respondent believes the beach needs a cleanup. All surveys were conducted by a first interviewer who did not live in the same neighborhood as the respondents. After completing the survey, a second interviewer joined, announcing whether they were from the neighborhood and inviting the respondent to join the clean-up. The interviewers followed a script to make sure they identically conveyed the message to all participants. The script differs between the treatment and control groups in that the interviewers referred to themselves either as neighbors or people from outside. After introducing themselves (“I live {name of the neighborhood}”, the interviewers provided information about the event and flyers for sharing with neighbors (see script and flyers in S4 File). They concluded with “If you are interested {we/me, as your neighbor} would be happy to see you next week!” Flyers provided by neighbor recruiters included the additional phrase, “Come and join your neighbors!” while flyers provided by community outsiders did not. The interviewer also encouraged the study participants to invite others outside their immediate family but within the same neighborhood to participate in the cleanup event. The initial purpose was to explore variations in the ability to mobilize neighbors across the different groups. However, very few respondents actually invited their neighbors (11 individuals recruited their neighbors in La Marsa, 3 in Le Kram and 5 in La Marsa).

Finally, the interviewers collected phone numbers to call the participants to remind them about the event and to follow up with the heads of households who did not show up at the cleanups (see consent forms in S4 File). After low participation in the pilot study, we decided to remind our respondents about the events. The interviewers said they would be reminded a few days before the cleanups. When the interviewers called them, they also asked whether they still intended to participate in the cleanups. This is a deviation from the initial study protocol. We added this question to capture planned participation before the cleanups.

We also provided positive incentives for the participants. The interviewers announced small lottery prizes beforehand as gift cards (worth 30,000 TND/ 9 USD), and they announced the winners at the events. We also provided water and trash bags to all participants. The clean-up events were organized on two Sundays—days off in Tunisia—about a week after the initial recruitment of the study participants took place. The enumerators called the participants to remind them about the events 2–3 days before the cleanups, and they asked whether they intended to come. Interviewers also ran a follow-up survey with all event participants and a phone survey with those who did not join the events.

### Experimental analysis

I estimate the average treatment effect (ATE) of neighbors’ social pressure on participation and the intentions to participate. I use an ordinary least square (OLS) estimator with the assignment to a neighbor recruiter as the treatment indicator. The outcomes are binary, indicating whether respondents reported that they intend to participate/ participated in the cleanup equals “1” and “0” otherwise.

Samples are balanced regarding gender, education, and employment status across both conditions—those recruited by a community outsider and those by a neighbor recruiter as presented in Table 1. However, respondents in the neighbor recruiter condition are, on average, older and more likely to be poor than those in the outsider recruiter condition. I control for these individual-level characteristics in the analysis to increase the precision of my estimates.

**Table 1:** Balance test and sample descriptive statistics.

	Community Outsider Recruiter	Neighbor Recruiter	p-value	Chi2
Gender				
Female	202 (47.06)	232 (52.94)	0.864	0.0295
Male	360 (46.54)	405 (53.46)		
Age				
18–29	241 (55.40)	194 (44.60)	0.000	23.0478
30–39	116 (45.14)	141 (54.86)		
40–49	87 (43.07)	115 (56.93)		
50–59	64 (39.51)	98 (60.49)		
60+	52 (37.14)	88 (62.86)		
Education				
No formal	4 (30.77)	9 (69.23)	0.122	5.7891
(Some) Primary	50 (39.37)	77 (60.63)		
(Some) Secondary	254 (49.51)	259 (50.49)		
Tertiary (University or other)	238 (46.03)	279 (53.97)		
Employment Status				
Employed	301 (47.48)	333 (52.52)	0.608	0.2624
Unemployed	252 (45.99)	296 (54.01)		
Wealth Status				
Poor	254 (42.19)	348 (57.81)	0.000	13.0401
Wealthy	266 (53.09)	235 (46.91)		

Note: Total numbers. Percentages are provided in brackets.

In the baseline survey, (see S8 File), we first asked the respondents about their general perception situation of solid waste in their neighborhood: “How much are public spaces like parks, fields or beaches, etc., around your neighborhood in need of cleanup? <1> not at all, <2> not much, <3> somewhat, <4> very much, <98> Don’t know/Refuse to answer”. On average, 91 percent of the respondents think that public spaces are somewhat or very much in need of a cleanup. Results vary significantly across the three neighborhoods with 85 percent in the wealthy neighborhood compared to 94 percent in the poor and mixed neighborhood (chi2(4)-test = 39.12, p = 0.000). Moreover, respondents in all three neighborhoods largely agree that the specific beach in their neighborhood requires a cleanup (91 percent in poor, 94 in mixed, and 90 percent in wealthy neighborhoods, chi2(2)-test = 5.56, p = 0.06). Almost 90 percent of the respondents reported coming to the beach in the future. Results vary significantly across neighborhoods with 84 percent in the poor neighborhoods, 87 percent in the mixed, and 95 percent in the wealthy neighborhood (chi2(2)-test = 26.74, p = 0.000).

Sixty-one percent, on average, fully agreed that a trashed environment represents a health risk to them and their families (see S12 Table for the responses by neighborhood). Responses were measured on a 10-point scale. A Wilcoxon signed-rank test indicates that agreement with the statement was significantly higher for respondents in the poor neighborhood compared to those in the mixed (z = 6.691, p<0.001) and significantly lower for respondents of the mixed neighborhood as compared to the wealthy neighborhood (z = -7.587, p<0.001). Differences between the poor and wealthy neighborhoods are not significant. Thus, I find evidence that respondents are generally aware of the problem and some associated risks. Most respondents also consider the beach a place they would want to visit.

The survey also asked about social norms to keep public spaces clean and, more generally, to engage in community activities. First, we asked about descriptive norms, which refer to what one would expect others to do, and second, about injunctive or moral norms, which refer to what someone ought to do [27, 28]: “When there are collective activities in this neighborhood, like cleaning a park or helping a family in need, do you think most of your neighbors help, more than half help, less than half help, almost none help? (<1> almost none, <2> less than half, <3> more than half, <4> most, <98> Don’t Know/Refuse to answer).” Findings on descriptive norms of engagement are mixed: Around 50 percent of the respondents (n = 1,199) report that they believe almost nobody or less than half of the people would join a neighborhood event (see S9 Table). These results already point to a potential coordination problem as people cannot expect their neighbors to join in an activity that requires many people to participate to succeed. Yet, unlike expected after running qualitative interviews in these neighborhoods in 2019, respondents in the wealthy neighborhood have more positive beliefs about their neighbors, with 48 percent reporting that most neighbors or almost everyone would join, compared to only 38 percent in the poor and 39 percent in the mixed neighborhood (chi2(4) = 24.81, p = 0.000). Moreover, sixty-two percent of the respondents in our baseline survey responded that fully agree that it is their civic duty to keep public spaces clean (see S10 Table, responses were measured on a 10-point scale). A Wilcoxon signed-rank test shows that agreement with the statement was significantly higher for respondents in the poor neighborhood compared to those in the mixed (poor = mixed, z = 2.526, p<0.05) and wealthy neighborhoods (z = 4.375, p<0.001). Differences between mixed and wealthy neighborhoods are not significant. Thus, we may infer from the answers that moral norms of engagement in civic activities exist within the neighborhoods. Around 40 percent of the respondents in each neighborhood agree that neighborhood initiatives lead to good outcomes (see S11 Table). Results vary significantly across neighborhoods with 71 percent in the poor, 58 percent in the mixed, and 74 percent in the wealthy neighborhood (chi2(4)-test = 69.88, p = 0.000). Yet I find mixed evidence for whether people believe that others would join the activity. These findings suggest citizens may face a classical social dilemma or coordination problem [1]. While participating in these activities is morally desirable, they cannot expect their neighbors to join and thus do not engage. I also asked several questions that aimed to measure community social ties. On average, 60 percent of the respondents report knowing some or most people in their neighborhood. This varies significantly between 51 percent in the poor and 64 percent in the mixed and wealthy neighborhoods (chi2(2) = 19.38, p = 0.000). Approximately 29 percent report that most or some of their neighbors are related to them (25 percent in the poor, 23 percent in the mixed, and 39 percent in the wealthy neighborhood, chi2(2) = 30.99, p = 0.000), and 55 percent report that some or most of their friends live in the neighborhood (51 percent in the poor, 49 percent in the mixed, and 64 percent in the wealthy neighborhood, chi2(2) = 19.16, p = 0.000), These results show that social ties were strongest among neighbors in the wealthy neighborhood. Lastly, I find that, on average, people have lived in the neighborhood for over 16 years (13 years in Le Kram, 14 years in La Goulette, and 23 years in La Marsa).

### Statistical results

In the following, I first discuss the findings for the entire sample before presenting the neighborhood analysis. I present the participation rates and numbers of recruited heads of households in S1 Table. I differentiate between intended participation—measured by a survey question asked over the phone three to two days before the event—and actual participation. Intended participation among respondents we reached through their phones is very high in the total sample. Ninety-two percent of the respondents confirmed their participation a few days before the events. Of the initial 1,199 heads of household, 252 (91 in La Marsa, 90 in La Goulette, and 71 in Le Kram) could not be reached. By contrast, actual participation is comparably low. On average, only 3 percent of the initially recruited heads of household participate in the neighborhood cleanups.

**Fig 1:**
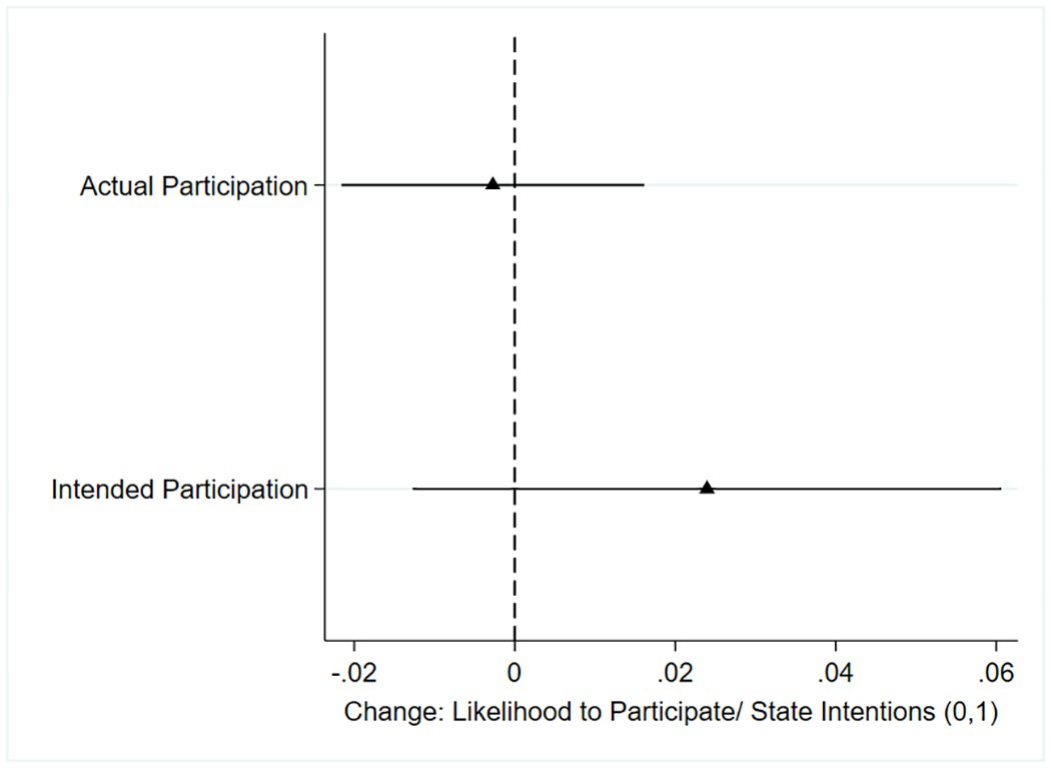
Average treatment effects using actual participation as DVs (95% CIs). Based on the OLS regression model with individual-level controls for age and wealth to deal with the imbalance between the treatment and placebo groups. See S7 and S8 Tables for regression Tables and S3 and S4 Tables without controls. Predicted probabilities are reported in S5 Table.

Fig 1 shows the point estimates of the average treatment effects for actual and intended participation as dependent variables. I do not find evidence that neighbor recruiters’ social pressure increases the likelihood of participating in the cleanups or the intentions to do so. Although participation rates are slightly higher for those respondents whom a neighbor recruited, the difference between the two treatment groups is not statistically significant for any of the standard non-parametric tests (see S2 Table). In addition, the chi2-test and Fischer’s exact test results suggest no statistically significant difference between being invited by a neighbor or a community outsider.

**Fig 2:**
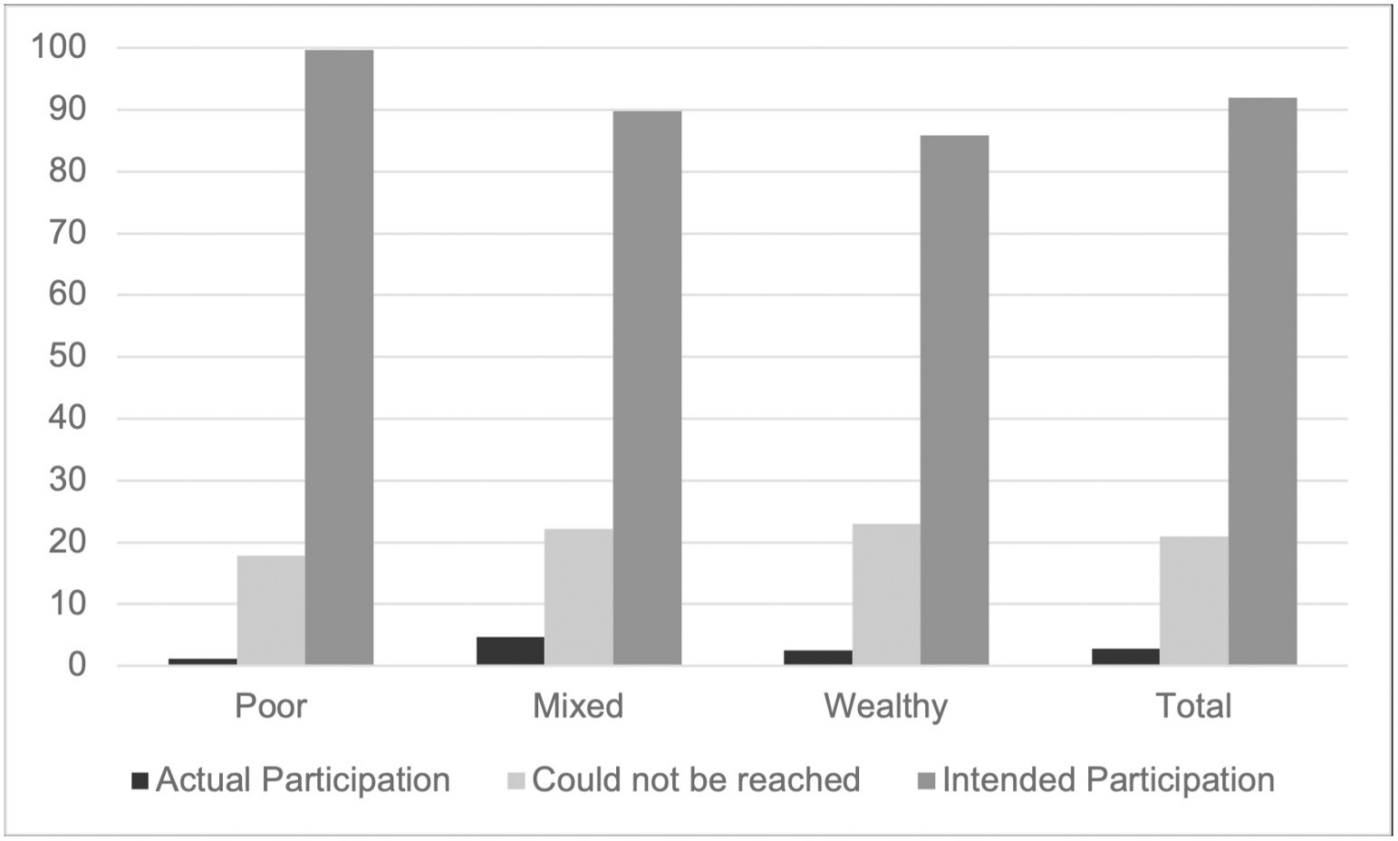
Intended vs actual participation in the cleanups by neighborhood (Percentages). Total numbers are reported in S1 Table. “Could not be reached” refers to study participants who did not pick up their phone when the interviewer called to ask about their intentions.

Regarding my first expectation, the study fails to reject the null hypothesis, which suggests that neighborly social pressure did not enhance collective action. However, due to low participation rates in the cleanups (see Fig 2), these suggestive null findings on the impact of social pressure on intended participation warrant cautious interpretation.

**Fig 3:**
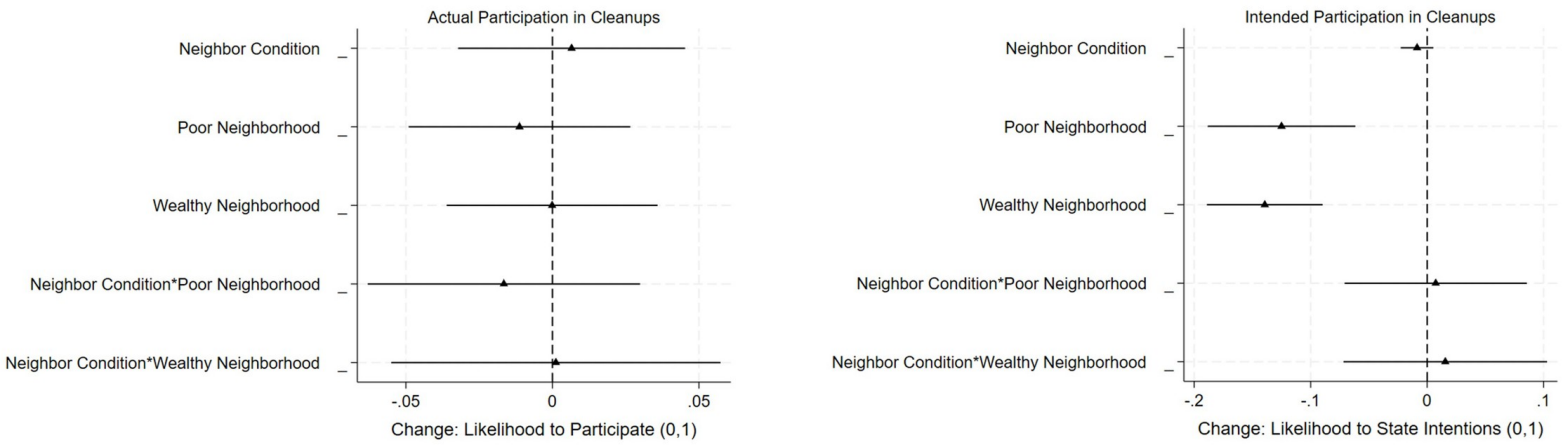
Average treatment effects using actual and intended participation as DVs and interaction between neighborhood context and social pressure (95% CIs). Based on the OLS regression model with individual-level controls for age and wealth to deal with the imbalance between the treatment and placebo groups.

Power analysis was performed before conducting the study and then again with the actual data from the experiment using the Declared Design Package in R to rule out that I failed to reject the null hypothesis when there was an effect (see S1 File). The experiment is sufficiently powered to detect medium-size treatment effects on cleanup participation (ATE = 0.05), which were found in previous neighbor canvasser experiments [3]. However, it is not well-powered to detect only small effect sizes and the impact on intentions to participate separately by neighborhood. We could not reach all participants by phone to ask about their intentions to participate. Therefore, our initial n = 1200 drops to n = 947 (see S1 Table). To test my second expectation (H2) that social pressure should have a stronger effect on civic participation in homogeneously poor neighborhoods as compared to wealthy or socioeconomically mixed neighborhoods, I run interactions between neighborhood context and social pressure on actual and intended participation (see Fig 3). The interaction coefficient is insignificant in both models, indicating that social pressure on participation did not significantly vary across the three communities with the mixed neighborhood as the baseline. Thus, I do not find support for H2.

Moreover, I also find descriptive evidence that reported intentions to participate are significantly higher at p<0.001 in poor neighborhoods than in wealthy and mixed neighborhoods (see S6 Table). By contrast, when comparing actual participation across the three neighborhoods, I do not find that the higher intentions to participate among the poor also translate into actual behavior. Indeed, participation is somewhat higher in the mixed and wealthy neighborhoods than in the poor neighborhood. Yet, the coefficients are only significant at p<0.1.

In the following, I will discuss potential reasons for the low participation rates in the cleanups and a few design-based limitations of this study that should be addressed in future research. First, one of the limitations of the study stems from the low participation rates in the cleanups. Crucially, the study may have faced ceiling and floor effects as high intentions and low participation rates make it difficult to detect changes related to social pressure.

Second, to better understand people’s reasons for abstaining, interviewers called the study participants after the events. On the phone, most respondents named one of the following reasons: they had other plans that day, were working, or had forgotten about the event (see S13 Table). Yet, except for time constraints, we could also consider other reasons people abstain.

Third, this brings me to one of the design-based limitations of the study. The pre-treatment survey asked the question of how many people would participate in a neighborhood initiative. The fact that it was asked before respondents were invited to the cleanups may have primed respondents in both the neighbor and outsider contact conditions to think about neighborly relations. Thus, although the survey question was designed to capture community coordination or bandwagoning, not neighborly social pressure, I cannot rule out that this has biased the results.

Moreover, respondents may not have personally known the recruiter in the neighbor condition, and thus, the social pressure treatment may have been weak. Of the 34 respondents who joined the cleanups, 47 percent of our respondents in the treatment condition reported that they know the recruiter personally compared to only 31 percent who know their recruiter in the community outsider treatment group. The relatively high percentage of cleanup participants who knew the outsider interviewer may stem from the fact that all interviewers were recruited from the greater Tunis area. It may also indicate that citizens are more likely to participate if they know the recruiter personally—independent of whether the person lives in the same neighborhood. However, as data was only available for those who joined the cleanups, the numbers cannot provide evidence of how many people knew the recruiters in the treatment and placebo groups. Moreover, recruiters in this study were 29 years old, on average. In comparison, most study participants are between 30–39 years old, which does not indicate any age imbalance between the two groups. Yet, in previous studies by Sinclair and colleagues [2, 3], the fact that canvassers introduced themselves as neighbors—as they did in this study—appeared to have induced high levels of social pressure on the individual. Therefore, we have good reason to expect that when our interviewers introduced themselves as neighbors, this created social pressure on the individuals.

Lastly, there is no reason to believe that Tunisians would be less responsive to social pressure from their neighbors than citizens of Western industrialized countries like the US, in which most previous literature was conducted. Findings from the baseline survey, as discussed in the analysis section, suggest that respondents in all three neighborhoods reported social ties to their neighbors. Respondents in the wealthy neighborhoods reported the strongest ties to their neighbors compared to those living in the poor and wealthy neighborhoods. On average, they have also lived in the neighborhood for longer (23 years) compared to the respondents in the poor and mixed neighborhoods. Thus, I do not find reason to believe that respondents simply do not care about being invited by their neighbors to participate in an event.

## Discussion and conclusion

Although I could not confirm my initial hypothesis, this study significantly contributes to the field. Most importantly, the findings question how far results on individually based forms of engagement—such as recycling and voting—hold when looking at other forms of civic and political engagement. They suggest that the mobilization for more time-intensive, socially based forms of action that require the engagement of many community members to be successful may not work in similar ways as, for example, voting. In particular, I find suggestive evidence that communities may face coordination problems.

Moreover, like many Get-Out-The-Vote Studies in Western democracies [2, 4, 9], the study has focused on social pressure by “ordinary” citizens. However, state and non-state leaders are central in mobilizing for community engagement in many countries in the global south [29, 30]. For example, work on local leaders in North Africa has emphasized the role of mosques and Islamist organizations in providing public goods and mobilizing voters [31, 32]. Future work should address whether local leaders like majors and religious dignitaries can be better mobilizers than neighbors and citizens without formal or informal leadership positions.

Interestingly, poor respondents are particularly likely to report that they intend to participate while being least likely to join the cleanups. Future work should explore these differences across socioeconomic groups to understand whether the poor are more likely to respond in a socially desirable way or whether my findings provide evidence for a “good intention gap” [33] among the poor. However, disentangling these two potential explanations would require measuring stated vs. actual intentions.

Importantly, my findings suggest that even where moral norms of engagement and general awareness of the problem exist, these may not be enough to spur participation when people do not expect their neighbors to join the activity. Thus, in line with some other recent work, my findings suggest that social pressure from neighbors may increase participation in collective action only in contexts where descriptive norms of engagement are already entrenched in society [2, 34] or when individuals share social ties with activists [35]. Moreover, some earlier work shows that descriptive voting norms are more strongly correlated with the voting behavior of the poor than the wealthy [14]. Future work should further investigate this variation in the responsiveness to social norms of engagement across the different socioeconomic groups.

If anything, social norms are more entrenched within the moderately conservative society of Tunisia. However, social norms may also be more difficult to change in this context. Previous work shows that Tunisians have developed a strong sense of local belonging following decades of political repression [36]. Thus, more work should explore the conditions under which local social norms can be changed, paying particular attention to variation across countries with different cultural and historical contexts.

Thus, although the study faces some design-based limitations that should be addressed in future research, I believe that the findings are equally important to scholars and policymakers. More research is needed to examine the role of social norms in communities to understand when these norms translate into collective action. Moreover, these findings show that campaigns that solely aim to increase the general awareness of environmental pollution may fail to reach active engagement in environmental protection and collective action. Instead, policymakers should design longer interventions to foster social norms of engagement in the communities.

## Supporting information

S1 Table Recruitment and intended vs actual participation in the cleanups by neighborhood. (DOCX)

S2 Table Descriptive statistics and nonparametric test statistics. (DOCX)

S3 Table Average treatment effects with actual participation without controls. (DOCX)

S4 Table Average treatment effects with intended participation without controls. (DOCX)

S5 Table Predicted probabilities based on logistic regression and actual participation as dependent variable. (DOCX)

S6 Table Differences in actual and intended participation across neighborhoods. (DOCX)

S7 Table Average treatment effects with actual participation and individual-level controls for poverty and age. (DOCX)

S8 Table Average treatment effects with intended participation and individual-level controls for poverty and age. (DOCX)

S9 Table How many people would participate in a neighborhood initiative (descriptive norm of engagement). (DOCX)

S10 Table Keeping public spaces clean is a civic duty by neighborhood (percent). (DOCX)

S11 Table Would a neighborhood initiative lead to good outcomes for the community? (DOCX)

S12 Table Waste as a health risk. (DOCX)

S13 Table Phone survey participants and reasons to abstain. (DOCX)

S1 File Power analysis. (PDF)

S2 File Pre-Analysis plan. (PDF)

S3 File Inclusivity in global research checklist. (PDF)

S4 File Consent forms and flyers. (PDF)

S5 File Neighborhood selection and sampling method. (PDF)

S6 File Hypotheses testing. (PDF)

S7 File IRB statement. (PDF)

S8 File Baseline survey. (PDF)
