# Supplementary material for: Neighborly social pressure and collective action: Evidence from a field experiment in Tunisia
Source: PLoS One. 2024 Jul 19;19(7):e0304269. doi: 10.1371/journal.pone.0304269 (PMC11259251; doi:10.1371/journal.pone.0304269)
Supplement: S1 Table — (DOCX) [file pone.0304269.s001.docx]

S1 Table. Recruitment and Intended vs Actual Participation in the Cleanups by Neighborhood

|  | Poor Neighborhood | Socioeconomically Mixed Neighborhood | Wealthy Neighborhood | Total |
| --- | --- | --- | --- | --- |
| Recruited HoH | 399  (100) | 405  (100) | 395  (100) | 1199 (100) |
| Intended Participation (Percent of those reached) | 327  (99.70) | 283  (89.84) | 261  (85.86) | 871  (91.97) |
| Intended not to Participate (Percent of those reached) | 1  (0.30) | 32  (10.16) | 43  (14.14) | 76  (8.03) |
| Not reachable via Phone | 71  (17.79) | 90  (22.22) | 91  (23.04) | 252 (21.02) |
| Actual Participation | 5  (1.25) | 19  (4.69) | 10  (2.53) | 34  (2.8) |

Note: Percentages in parentheses.
