# Supplementary material for: Neighborly social pressure and collective action: Evidence from a field experiment in Tunisia
Source: PLoS One. 2024 Jul 19;19(7):e0304269. doi: 10.1371/journal.pone.0304269 (PMC11259251; doi:10.1371/journal.pone.0304269)
Supplement: S2 Table — (DOCX) [file pone.0304269.s002.docx]

S2 Table. Descriptive Statistics and Nonparametric Test Statistics

|  | Community Outsider^§^ | Neighbor^§^ | Pearson Chi2 (p-value) | Fisher´s Exact (one sided) |
| --- | --- | --- | --- | --- |
| Actual Participation | 13 (2.31) | 21 (3.30) | 1.048 (0.306) | 0.384 (0.198) |
| Intended Participation (of those reached) | 400 (71.17) | 471 (73.94) | 4.0221 (0.045) | 0.055 (0.030) |

Note: ^§^Absolut numbers reported for Treatment Groups. Percentages are presented in parentheses.
