# Supplementary material for: Neighborly social pressure and collective action: Evidence from a field experiment in Tunisia
Source: PLoS One. 2024 Jul 19;19(7):e0304269. doi: 10.1371/journal.pone.0304269 (PMC11259251; doi:10.1371/journal.pone.0304269)
Supplement: S3 Table — (DOCX) [file pone.0304269.s003.docx]

S3 Table. Average Treatment Effects with Actual Participation without Controls

|  | Model (1)  Actual Participation | Model (2)  Actual Participation Le Kram | Model (3)  Actual Participation La Goulette | Model (4)  Actual Participation La Marsa |
| --- | --- | --- | --- | --- |
| Treatment | 0.0098  (0.0120) | -0.0039  (0.0119) | 0.0242  (0.0216) | 0.0112  (0.0172) |
| Constant | 0.0231**  (0.0039) | 0.0152  (0.0097) | 0.0321*  (0.0169) | 0.0219**  (0.0095) |
| Observations | 1199 | 399 | 405 | 395 |
| R2 | 0.0009 | 0.0003 | 0.0031 | 0.0011 |

Note: *p<0.1 **p<0.05 ***p<0.01. Based on OLS regression. Standard errors in parentheses. Clustered standard errors on the neighborhood level in Model (1).
