# Supplementary material for: Neighborly social pressure and collective action: Evidence from a field experiment in Tunisia
Source: PLoS One. 2024 Jul 19;19(7):e0304269. doi: 10.1371/journal.pone.0304269 (PMC11259251; doi:10.1371/journal.pone.0304269)
Supplement: S4 Table — (DOCX) [file pone.0304269.s004.docx]

S4 Table. Average Treatment Effects with Intended Participation without Controls

|  | Model (1)  Intended Participation | Model (2)  Intended Participation Le Kram | Model (3)  Intended Participation La Goulette | Model (4)  Intended Participation La Marsa |
| --- | --- | --- | --- | --- |
| Treatment | 0.036*  (0.018) | -0.005  (0.006) | 0.006  (0.036) | 0.027  (0.043) |
| Constant | 0.901***  (0.013) | 1.000***  (0.005) | 0.892***  (0.028) | 0.853***  (0.024) |
| Observations | 947 | 328 | 316 | 303 |
| R2 | 0.004 | 0.002 | 0.000 | 0.001 |

Note: *p<0.1 **p<0.05 ***p<0.01. Based on OLS regression. Standard errors in parentheses. Clustered standard errors on the neighborhood level in Model (1). Respondents who could not be reached via phone have been dropped from the analysis.
