# Supplementary material for: Neighborly social pressure and collective action: Evidence from a field experiment in Tunisia
Source: PLoS One. 2024 Jul 19;19(7):e0304269. doi: 10.1371/journal.pone.0304269 (PMC11259251; doi:10.1371/journal.pone.0304269)
Supplement: S5 Table — (DOCX) [file pone.0304269.s005.docx]

S5 Table. Predicted Probabilities based on Logistic Regression and Actual Participation as Dependent Variable

|  | Margin | SE | P-value | 95% Conf. Interval | 95% Conf. Interval |
| --- | --- | --- | --- | --- | --- |
| Community Outsider | .0219884 | .0041915 | 0.000 | .0137732 | .0302037 |
| Neighbor | .0250146 | .0129199 | 0.053 | -.000308 | .0503373 |

Note: Predicted probabilities based on logistic regression model with controls for age, gender, education, employment status held at the median level. Standard errors are clustered at the neighborhood level.
