# Supplementary material for: Neighborly social pressure and collective action: Evidence from a field experiment in Tunisia
Source: PLoS One. 2024 Jul 19;19(7):e0304269. doi: 10.1371/journal.pone.0304269 (PMC11259251; doi:10.1371/journal.pone.0304269)
Supplement: S6 Table — (DOCX) [file pone.0304269.s006.docx]

S6 Table. Differences in Actual and Intended Participation across Neighborhoods

|  | Model (1)  Actual Participation | Model (2)  Intended Participation |
| --- | --- | --- |
| Treatment | 1.063  (0.429) | 0.980  (0.266) |
| Neighborhood (Poor as baseline) |  |  |
| Mixed | 2.981*  (1.761) | 0.021***  (0.022) |
| Wealthy | 3.103*  (1.923) | 0.020***  (0.021) |
| Individual Controls |  |  |
| Age |  |  |
| 30-39 | 1.141  (0.712) | 1.718  (0.623) |
| 40-49 | 1.433  (0.906) | 1.947  (0.851) |
| 50-59 | 2.226  (1.131) | 1.451  (0.579) |
| 60+ | 1.989  (1.280) | 1.539  (0.658) |
| Poor | 1.684  (0.720) | 1.237  (0.336) |
| Constant | 0.006***  (0.004) | 220.874***  (223.973) |
| Observations | 1,101 | 869 |
| Pseudo R2 | 0.043 | 0.128 |
| Log Likelihood | -124.8484 | -212.35028 |

Note: *p<0.1 **p<0.05 ***p<0.01. Logistic regression. Odds ratios are reported.
