# Supplementary material for: Neighborly social pressure and collective action: Evidence from a field experiment in Tunisia
Source: PLoS One. 2024 Jul 19;19(7):e0304269. doi: 10.1371/journal.pone.0304269 (PMC11259251; doi:10.1371/journal.pone.0304269)
Supplement: S7 Table — (DOCX) [file pone.0304269.s007.docx]

S7 Table. Average Treatment Effects with Actual Participation and Individual-Level Controls for Poverty and Age

|  | Model (1)  Actual Participation^1^ | Model (2)  Actual Participation Le Kram | Model (3)  Actual Participation La Goulette | Model (4)  Actual Participation La Marsa |
| --- | --- | --- | --- | --- |
| Treatment | -0.027  (0.008) | -0.011  (0.011) | 0.006  (0.020) | 0.014  (0.019) |
| Age (18-29 as baseline) |  |  |  |  |
| 30-39 | 0.006  (0.027) | 0.015  (0.015) | 0.047  (0.033) | -0.044*  (0.023) |
| 40-49 | 0.010  (0.017) | 0.017  (0.015) | 0.036  (0.033) | -0.023  (0.028) |
| 50-59 | 0028  (0.013) | 0.031  (0.019) | 0.046  (0.033) | 0.003  (0.029) |
| 60+ | 0.022  (0.015) | 0.030  (0.019) | 0.040  (0.033) | -0.006  (0.036) |
| Poor | 0.011  (0.005) | 0.005  (0.011) | 0.015  (0.022) | 0.015  (0.019) |
| Constant | 0.011  (0.013) | 0.004  (0.011) | -0.010  (0.028) | 0.032**  (0.015) |
| Observations | 1101 | 383 | 369 | 349 |
| R2 | 0.006 | 0.015 | 0.010 | 0.015 |

Note: *p<0.1 **p<0.05 ***p<0.01. Based on OLS regression. Standard errors in parentheses. ^1^Clustered standard errors on the neighborhood level in Model (1).
