# Supplementary material for: Neighborly social pressure and collective action: Evidence from a field experiment in Tunisia
Source: PLoS One. 2024 Jul 19;19(7):e0304269. doi: 10.1371/journal.pone.0304269 (PMC11259251; doi:10.1371/journal.pone.0304269)
Supplement: S8 Table — (DOCX) [file pone.0304269.s008.docx]

S8 Table. Average Treatment Effects with Intended Participation and Individual-Level Controls for Poverty and Age

|  | Model (1)  Actual Participation^1^ | Model (2)  Actual Participation Le Kram | Model (3)  Actual Participation La Goulette | Model (4)  Actual Participation La Marsa |
| --- | --- | --- | --- | --- |
| Treatment | 0.024  (0.018) | -0.006  (0.007) | -0.003  (0.038) | -0.004  (0.045) |
| Age (18-29 as baseline) |  |  |  |  |
| 30-39 | 0.014  (0.024) | 0.007  (0.009) | 0.103*  (0.062) | 0.042  (0.053) |
| 40-49 | 0.025  (0.027) | 0.008  (0.009) | 0.117*  (0.063) | 0.048  (0.068) |
| 50-59 | -0.008  (0.030) | 0.009  (0.012) | 0.081  (0.061) | 0.044  (0.071) |
| 60+ | -0.002  (0.058) | 0.008  (0.012) | 0.156**  (0.062) | -0.132*  (0.080) |
| Poor | 0.027  (0.022) | -0.006  (0.007) | 0.008  (0.041) | 0.052  (0.044) |
| Constant | 0.887***  (0.059) | 1.000***  (0.007) | 0.789***  (0.054) | 0.832***  (0.036) |
| Observations | 869 | 314 | 284 | 271 |
| R2 | 0.007 | 0.009 | 0.025 | 0.025 |

Note: *p<0.1 **p<0.05 ***p<0.01. Based on OLS regression. Standard errors in parentheses. ^1^Clustered standard errors on the neighborhood level in Model (1). Respondents who could not be reached via phone have been dropped from the analysis.
