# Supplementary material for: Neighborly social pressure and collective action: Evidence from a field experiment in Tunisia
Source: PLoS One. 2024 Jul 19;19(7):e0304269. doi: 10.1371/journal.pone.0304269 (PMC11259251; doi:10.1371/journal.pone.0304269)
Supplement: S9 Table — (DOCX) [file pone.0304269.s009.docx]

S9 Table. How many People would Participate in a Neighborhood Initiative (Descriptive Norm of Engagement)

|  | **Poor**  **Neighborhood** | **Mixed Neighborhood** | **Wealthy Neighborhood** |
| --- | --- | --- | --- |
| Almost none | 43 (10.78) | 67 (16.54) | 38 (9.62) |
| Less than half | 181 (45.36) | 143 (35.31) | 122 (37.20) |
| More than half | 98 (24.56) | 96 (23.70) | 141 (35.70) |
| Most | 55 (13.78) | 60 (14.81) | 48 (12.15) |
| Do not know/ Refuse to answer | 22 (5.51) | 39 (9.63) | 46 (11.65) |
| Total | 399 (100) | 405 (100) | 395 (100) |

Note: Absolut numbers reported. Percentages in parentheses. Answers to the following survey question: “When there are collective activities in this neighborhood, like cleaning a park or helping a family in need, do you think most of your neighbors help, more than half help, less than half help, almost none help? (<1> almost none, <2> less than half, <3> more than half, <4> most, <98> Don't Know/Refuse to answer).”
