# Supplementary material for: Neighborly social pressure and collective action: Evidence from a field experiment in Tunisia
Source: PLoS One. 2024 Jul 19;19(7):e0304269. doi: 10.1371/journal.pone.0304269 (PMC11259251; doi:10.1371/journal.pone.0304269)
Supplement: S10 Table — (DOCX) [file pone.0304269.s010.docx]

S10 Table. Keeping Public Spaces clean is a Civic Duty by Neighborhood (percent)

|  | **Poor**  **Neighborhood** | **Mixed Neighborhood** | **Wealthy Neighborhood** |
| --- | --- | --- | --- |
| 0 | 9 (2.26) | 13 (3.21) | 19 (4.81) |
| 1 | 4 (1) | 8 (1.98) | 0 |
| 2 | 5 (1.25) | 9 (2.22) | 6 (1.52) |
| 3 | 1 (0.25) | 4 (0.99) | 5 (1.27) |
| 4 | 3 (0.75) | 8 (1.98) | 5 (1.27) |
| 5 | 28 (7.02) | 20 (4.94) | 41 (10.38) |
| 6 | 5 (1.25) | 12 (2.96) | 7 (1.77) |
| 7 | 10 (2.51) | 14 (3.46) | 14 (3.54) |
| 8 | 28 (7.02) | 40 (9.88) | 38 (9.62) |
| 9 | 28 (7.02) | 27 (6.67) | 47 (11.90) |
| 10 | 278 (69.67) | 250 (61.73) | 213 (53.92) |
| Total | 399 (100) | 405 (100) | 395 (100) |

Note: Absolut numbers reported. Percentages in parentheses. Responses to the following survey question are presented: “On a scale from 0 to 10 where 0 means not at all and 10 very much, how much do you agree with the following statements: Keeping public spaces (e.g., the beach, playgrounds, etc.) clean is a civic duty.”
