# Supplementary material for: Neighborly social pressure and collective action: Evidence from a field experiment in Tunisia
Source: PLoS One. 2024 Jul 19;19(7):e0304269. doi: 10.1371/journal.pone.0304269 (PMC11259251; doi:10.1371/journal.pone.0304269)
Supplement: S11 Table — (DOCX) [file pone.0304269.s011.docx]

S11 Table. Would a Neighborhood Initiative lead to Good Outcomes for the Community?

|  | **Poor**  **Neighborhood** | **Mixed**  **Neighborhood** | **Wealthy Neighborhood** |
| --- | --- | --- | --- |
| Not much at all | 28 (7.02) | 58 (14.32) | 20 (5.06) |
| Not much | 84 (21.05) | 108 (26.67) | 57 (14.43) |
| Somewhat much | 102 (25.56) | 67 (16.54) | 123 (31.14) |
| Very much | 182 (45.61) | 167 (41.23) | 169 (42.78) |
| Do not know/ Refuse to answer | 3 (0.75) | 5 (1.23) | 26 (6.58) |
| Total | 399 (100) | 405 (100) | 395 (100) |

Note: Absolut numbers reported. Percentages in parentheses. Responses to the following survey question are presented: “How much do you think that neighborhood initiatives or events lead to good outcomes for the neighborhood? <1> not much at all, <2> not much, <3> somewhat much, <4> very much, <98> Don’t know/Refuse to answer.”
