# Supplementary material for: Neighborly social pressure and collective action: Evidence from a field experiment in Tunisia
Source: PLoS One. 2024 Jul 19;19(7):e0304269. doi: 10.1371/journal.pone.0304269 (PMC11259251; doi:10.1371/journal.pone.0304269)
Supplement: S12 Table — (DOCX) [file pone.0304269.s012.docx]

S12 Table. Waste as a Health Risk

|  | **Poor**  **Neighborhood** | **Mixed Neighborhood** | **Wealthy Neighborhood** |
| --- | --- | --- | --- |
| 0 | 10 (2.51) | 14 (3.46) | 16 (4.05) |
| 1 | 5 (1.25) | 10 (2.47) | 3 (0.76) |
| 2 | 4 (1) | 12 (2.96) | 1 (0.25) |
| 3 | 1 (0.25) | 9 (2.22) | 3 (0.76) |
| 4 | 5 (1.25) | 11 (2.72) | 3 (0.76) |
| 5 | 23 (5.76) | 31 (7.65) | 10 (0.76) |
| 6 | 7 (1.75) | 25 (6.17) | 9 (2.28) |
| 7 | 10 (2.51) | 31 (7.65) | 7 (1.77) |
| 8 | 43 (10.78) | 49 (12.10) | 20 (5.06) |
| 9 | 16 (4.01) | 28 (6.91) | 47 (11.90) |
| 10 | 275 (68.92) | 185 (45.68) | 276 (69.87) |
| Total | 399 (100) | 405 (100) | 395 (100) |

Note: Absolut numbers reported. Percentages in parentheses. Responses to the following survey question are presented: “On a scale from 0 to 10 where 0 means not at all and 10 very much, how much do you agree with the following statements: Waste presents a health risk to you and your family.”
