# Supplementary material for: Neighborly social pressure and collective action: Evidence from a field experiment in Tunisia
Source: PLoS One. 2024 Jul 19;19(7):e0304269. doi: 10.1371/journal.pone.0304269 (PMC11259251; doi:10.1371/journal.pone.0304269)
Supplement: S13 Table — (DOCX) [file pone.0304269.s013.docx]

S13 Table. Phone Survey Participants and Reasons to Abstain

|  | **Poor**  **Neighborhood** | **Mixed**  **Neighborhood** | **Wealthy Neighborhood** |
| --- | --- | --- | --- |
| I was working that day | 17 (22.67) | 18 (26.87) | 24 (37.50) |
| I had other plans | 33 (44.00) | 29 (43.28) | 11 (17.19) |
| I had forgotten about the event | 16 (21.33) | 8 (11.94) | 7 (10.94) |
| Me or someone from my family was sick | 4 (5.33) | 8 (12.31) | 10 (15.63) |
| Number of Respondents | 75 | 65 | 64 |

Note: Respondents were called the week after the cleanups. Interviewers called them at least two times when they could not reach the respondents.
