# Supplementary material for: Neighborly social pressure and collective action: Evidence from a field experiment in Tunisia
Source: PLoS One. 2024 Jul 19;19(7):e0304269. doi: 10.1371/journal.pone.0304269 (PMC11259251; doi:10.1371/journal.pone.0304269)
Supplement: S1 File — (PDF) [file pone.0304269.s014.pdf]

Supplementary Material – S1

Power Analysis

Power Analysis in Stata Pre-Registration

I run a power analysis for for a test of two independent proportions with my treatment groups and participation as binary outcome.

Using Stata`s sampsi command, I calculate the sample size for the experiment. Previous Get-Out-The-Vote studies have found a robust effect size of 7-10 percentage points difference in vote turnout through canvassing (Sinclair et al. 2013). Similarly, Sinclair et al. (2013) find a 4-11 percentage point increase in voter turnout through local canvassing. I have assumed an effect size of 13 percentage points in the calculations below. Yet for the final calculation, the expected participation rate in both groups (among heads of households) will be based on findings from the pilot study. The alpha level is set to .05 according to standards in political science and the required level of power for this experiment is set to .80. I use a one-tail test as I expect the treatment to increase participation.

The calculation indicates that I would need to recruit at least 125 heads of households in a neighborhood in each group (treatment and placebo groups) to find a difference in the probability of participating by .15 with a power of .8 and an alpha of .05.

```
sampsi .25 .12, power(.8) onesided

Estimated sample size for two-sample comparison of proportions

Test H0: p1 = p2, where p1 is the proportion in population 1
                    and p2 is the proportion in population 2
Assumptions:

         alpha =   0.0500  (one-sided)
         power =   0.8000
            p1 =   0.2500
            p2 =   0.1200
         n2/n1 =   1.00

Estimated required sample sizes:

            n1 =      125
            n2 =      125
```

However, as we had very low participation rates of initially recruited heads of households (less than 10 percent), we decided to increase the number of respondents that were recruited for the first survey to 400 for each sample. This means that we have 200 respondents in the neighbor recruiter treatment and 200 in the community outsider recruiter group per neighborhood sample.

Power Analysis with Collected Data

I ran a power analysis with the data obtained from the experimental study to rule out that the experiment was underpowered to detect average treatment effects. I use the Declared Design online tool (link: https://eos.wzb.eu/ipi/DDWizard/) provided by the Evidence in Governance and Politics network (EGAP) to calculate power for different sample sizes. The online tool was not available at the time of the pre-registration of the experimental design, which is why I had performed the power analysis in STATA at the time.

Figure B1. Power Calculation using different Samples Sizes and Participation as DV

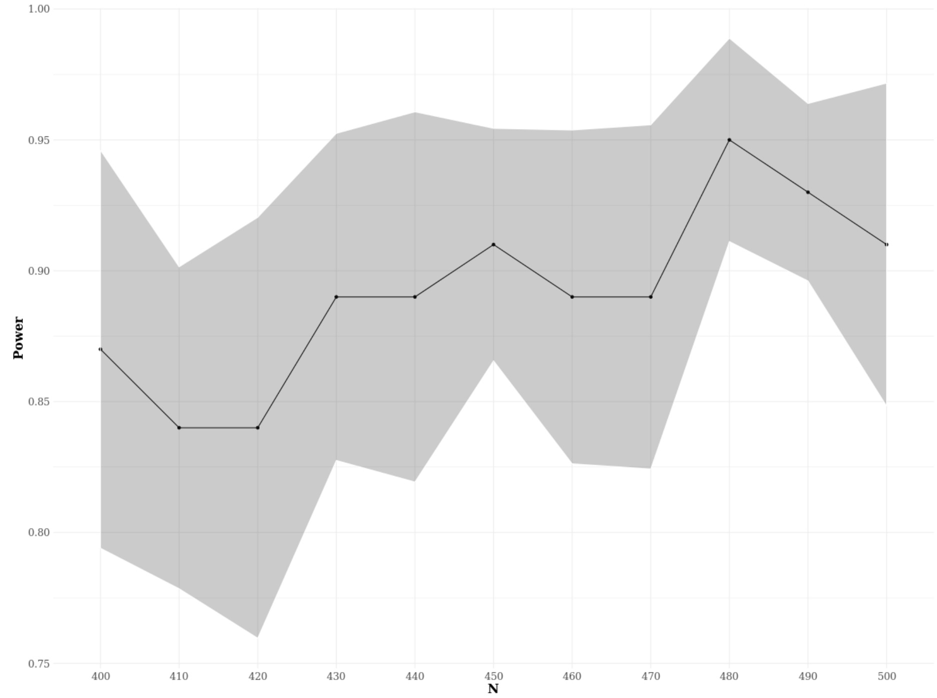

Note: The power analysis was calculated for ATE=0.05, and the control mean and standard deviations for the control and treatment groups were calculated using data from the actual experiment. Participation in the cleanup as the dependent variable. (n=400 for each neighborhood).

The power analysis shows that the experiment was significantly powered to detect possible average treatment effects on actual participation in each neighborhood (n=400). The estimated power for n=400 presented in Figure B1 mitigates concerns about power issues for the interpretation of the null findings in the experiment.

Figure B2. Power Calculation using different Samples Sizes and Intentions as DV

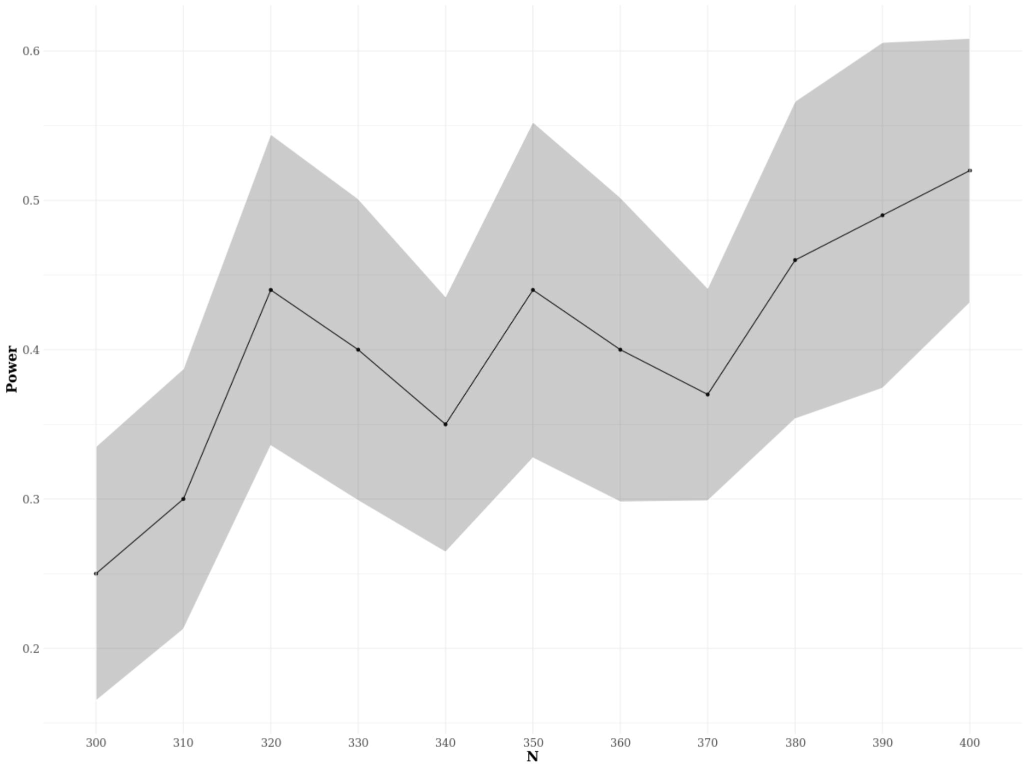

Note: The power analysis was calculated for ATE=0.05, and the control mean and standard deviations for the control and treatment groups were calculated using data from the actual experiment. Participation in the cleanup as the dependent variable. (n=400 for each neighborhood).

The power analysis for intentions to participate shows that the experiment was not sufficiently powered to detect treatment effects on the intentions to participate. The n dropped for all three neighborhoods from initially n=400 recruited study participants to n=328 in Le Kram, n=316 in La Goulette, and n=303 in La Marsa because we could not reach all our initial study participants through their phones.
