# Supplementary material for: Neighborly social pressure and collective action: Evidence from a field experiment in Tunisia
Source: PLoS One. 2024 Jul 19;19(7):e0304269. doi: 10.1371/journal.pone.0304269 (PMC11259251; doi:10.1371/journal.pone.0304269)
Supplement: S2 File — (PDF) [file pone.0304269.s015.pdf]

Pre-Analysis Plan

Local Context, Social Norms and Civic Participation

A Fieldexperiment in Tunisia

Introduction

Survey data from Tunisia (*anonymized*) demonstrates wide variation in civic engagement across individuals and communities of differing socioeconomic backgrounds. A nationally representative household survey of 3,659 respondents that was conducted in 2015 in Tunisia, reveals that among those who self-identify with the lower-class, 31 percent report helping their neighbors clean the neighborhood, compared to just about 20 percent of those who self-identified with the middle or with the upper class. Moreover, respondents who live in the poorer interior governorates of the country are 22 percentage points more likely to report engaging in neighborhood clean-ups compared to those in the wealthy coastal governorates. Even within the same governorate, responses vary between citizens living in homogenously poor municipalities, where 62 percent of the respondents report that they help their neighbor, and more mixed municipalities, where 5 percent of the respondents report that they help their neighbors.[1]

These findings point to the importance of socioeconomic background and context for community engagement. Yet, despite extensive previous research on mobilization and participation more generally, we still do not know exactly what drives community participation among the poor. Moreover, we lack a deeper understanding of how different social contexts affect individual and interpersonal behavior or which contexts are most favorable for encouraging community collective action, and why.

With this research project I aim to investigate the questions *when and why do citizens and, in particular, less-affluent individuals participate in collective action?* To begin to unpack the

[1] An analysis by reported social class shows that in Bou-Aarda, where 57 percent of the respondents identify as members of the lower class, 87 percent of respondents reported helping their neighbors compared to 31 percent of the middle class and 21 percent of the upper class. By contrast, only 20 percent of the respondents in Gaafour self-identified with the lower class and only 2 percent of these reported helping their neighbors to clean the neighborhood (compared to 5 percent of the middle class and 16 percent of the upper class).

answers to these questions, I consider both individual and community drivers of participation in a neighborhood clean-up initiative. I expect that part of the answer lies in the extent to which social norms surrounding obligations and reciprocity among neighbors vary across individuals and communities of differing socioeconomic backgrounds.

First, the research project builds a model of how social norms and obligations among neighbors contribute to participation in community initiatives, more generelly. Drawing on a rich body of literature, I employ potential mechanisms that translate social norms into actual behavior: 1) community sanctioning (e.g., Sinclair 2012) and 2) generalized social trust (e.g., Putnam 2005); 3) solidarity, or group attachment (Baldassarri 2015), and 4) reciprocity (Cialdini and Goldstein 2004; Gouldner 1960). In the literature, these moderators are widely discussed, but are rarely measured at the same time to unpack what is doing the work.

More specifically, I also expect the impact of local social norms and the community moderators on individual participation to vary by individual weath and social neighborhood context, measured as the socioeconomic composition and the density of social ties in the neighborhood. I expect some of these moderators to be activated only among poor or wealthy individuals. For example, I hypothesize that poor individuals rely more frequently on their neighbors for help which induces norms of reciprocity and social obligations (e.g., Piff 2010; Scott 1976; Kersting and Sperberg 2003), whereas wealthier individuals have been found to show higher levels of generalized social trust (Putnam 1993, 2000).

Finally, I expect social context to moderate the effect of individual poverty on participation. I differentiate between homogeneously poor neighborhoods in which lower-class individuals are in the majority, homogeneously wealthy neighborhoods in which upper-class respondents constitute the majority and socioeconomically mixed neighborhoods. My underlying assumption is that social ties should be denser in the homogeneously poor community but I also test this assumption and the density and strength of social ties in socioeconomically mixed and wealthy neighborhoods.

To empirically test my model, I propose to run a field experiment involving clean-up events and surveys in three different neighborhoods in Tunis. I purposively select a homogeneously poor neighborhood, in which lower-class individuals are in the majority, a homogeneously wealthy neighborhood and a socioeconomically mixed neighborhood, in which upper-class

respondents and lower-class respondents intermingle. Since I am unable to experimentally manipulate the socioeconomic status of an individual as well as the context in which he or she is living, I can only examine associations between these factors and the outcomes. Yet, the study experimentally tests what I believe to be an underlying mechanism: social pressure in the neighborhood. Specifically, I will try to activate social norms of engagement through invitations to participate by neighbors versus community outsiders. For a randomly selected half of our sample, a neighbor will recruit participants to a community clean-up (treatment group); for the other half of the sample, a person who is not from the area will recruit them (control group). We run pre- and post-treatment surveys with all participants in the experiment and phone surveys with those who did not show up at the actual clean-up event.

In this proposal, I first build a theoretical framework from the extant literature on how social norms translate into civic engagement at the community level and how it should vary between different socioeconomic groups and across neighborhood contexts. I offer an overview of work on the impact of social neighborhood context on civic and political behavior. A list of the hypotheses at the individual and community levels follows. Then, I introduce the field experiment to test my theoretical model.

Social Norms and Civic Engagement

This project lies at the nexus of three important strands of literature. The first strand seeks to understand what drives civic engagement more generally? The work reviewed in this subsection is mostly conducted in the West, examining correlations between reported participation and various mediators that are thought to translate social norms into participation in various forms of civic initiatives. I take this work and develop my own theoretical framework for understanding how social norms translate into civic engagement. The second strand of literature considers civic participation among the wealthy versus the poor, taking into consideration both how documented patterns of participation as well as how the often-cited mediators of social action may differ across both groups. Finally, a third literature suggests that contextual effects are an important consideration for community mobilization. As this study proposes to contribute to all three of these spheres, I provide an overview of each of these in this section.

The Activation of Social Norms and Their Translation into Civic Engagement

Within the last three decades, scholars have worked extensively on the role of social norms in motivating local civic participation—by which I mean participation in activities relating to the improvement of one's local community. For instance, a wide array of public opinion research spanning various types of civic activities demonstrates a robust correlation between strong social ties among community members and civic engagement. Other scholars employ experimental manipulations to causally demonstrate that the application of social pressure spurs participation. Both of these bodies of literature posit that the activation of social norms—generally accepted notions of proper and moral behavior—motivate their outcomes at least to some extent, yet, more work is needed to clarify how such norms translate into civic behavior. In this study, I combine these research methods to help clarify the relationship between social norms and civic engagement drawing on work from political science, sociology, and social psychology.

A vast body of work employing survey data correlates stronger community ties and norms with higher civic participation. One of the most famous of these studies is Robert Putnam's *Bowling Alone* in which he introduces social capital as the "connections among individuals—social networks and the norms of reciprocity and trustworthiness that arise from them" (Putnam 2000, 19) and links it with reported civic engagement in surveys across a variety of groups and activities over time.[2] In their seminal work, *Voice and Equality: Civic Voluntarism in American Politics,* Verba et al. (1995, chapters 4-5) analyze a survey of 15,000 participants elucidating how reported civic participation increases when requests come during face-to-face encounters with personal connections and that community engagement may be more socially gratifying when undertaken with friends. Pavlova and Silbereisen (2015) correlate support from significant others, including friends, family members, and other local community members with participation in civic and political life. Similarly, Tucker (1999) shows that higher numbers of frequent recyclers within a neighborhood are associated with higher

[2] The differentiation between civic and political modes of participation is not often clear throughout the body of work we consider in this literature review. Although we are interested in a case of civic engagement in neighborhood clean-up events, we think that the literature looking at political engagement is relevant for our work. Political and civic participation both involve collective action among members of a social group and are therefore likely to be affected similarly by social norms. Of course, there may be important differences between motivating participation in local elections compared to more remote, national elections that could be important, but such differentiation is not the focus of our study here though it may pose a limitation to the applicability of its findings.

collection set out rates. In a follow-up survey, respondents living on high performing streets report having been persuaded by their neighbors and being influenced by the high number of garbage bags their neighbors set out on the streets. The findings suggest that individuals orient their own behavior towards the actions of their neighbors which suggests that social pressure is activating their willingness to comply. All of these studies more generally indicate that social ties or pressure induce civic action. Yet, these are correlational outcomes as they do not experimentally manipulate social pressure to be able to test it as the underlying causal driver of participation.

Much of the experimental research on community participation provides empirical evidence that social pressure encourages individual participation in voting (e.g., Gerber et al. 2008; Sinclair 2012), recycling (e.g., Tucker 1999; Schultz 1999; Burn 1991), and other types of environmental action initiatives (e.g., Hoffman and High-Pippert 2010). Gerber, Green, and Larimer (2008) activate social pressure by sending mailings with real information of past voting records in the area, noting that after the upcoming statewide primary elections,[3] it would be reported whether the respondent voted or not in a mailing to their household or also to their neighbors compared to a control message that primed one's civic duty to vote. They find that revealing one's voting behavior to household members increases turnout by 5 percentage points, while publicizing this information to neighbors increased it by 8 percentage points. Although this work is important in demonstrating that social pressure can induce political engagement on a mass level, what are the mechanisms that explain this effect of social pressure on civic engagement?

Building upon this work, Sinclair (2012) sends door-to-door canvassers from within the neighborhood and compared them to canvassers from outside the area to test the effect of localized social pressure on voter turnout. She argues that local canvassers are more likely to interact with their recruitees in the future and, therefore, to be able to monitor as well as sanction their lack of compliance with social norms (Sinclair 2012, 37). She also cites work by Rosenstone and Hansen (1993, 23) positing that people seek to be "accepted, valued, and liked" by their neighbors and as such, want to meet their expectations, and strive to fulfill the obligations imposed by them. In another study, Schultz (1999) conducts a field experiment on

[3] Participation in these elections can be considered to be civic engagement as it pertained to "wide range of offices and proposals on the ballot, most of which were limited to counties, cities, and local districts" (Gerber et al. 2008, 36).

recycling in which individuals were randomly assigned to receive a plea, individual feedback, or group feedback regarding their behavior (frequency and number of recyclables set out). Individuals are significantly more likely to participate in curbside recycling when they receive individual and group feedback from neighbors than just information about recycling or a plea to recycle from researchers. Finally, a number of studies employing field experiments attempt to manipulate social pressure and thereby activate social norms to increase recycling. These studies typically find that people in places where neighbors are assigned as block leaders are significantly more likely to recycle when neighbors are assigned as block leaders than areas without such leaders (Nielsen & Ellington 1983), and in particular when the leaders establish personal contact with households (Hopper & Nielsen 1991; Burn 1991) compared to just informational brochures or bags being left at the door.

Yet, because these experimental studies did not employ surveys of their participants (although Hopper & Nielsen 1991 are an exception here),[4] while we can see the effects of the experimental conditions, we are unable to pinpoint the mechanisms driving civic engagement. Since social norms—and the various mechanisms cited for why their effectiveness (discussed more below)—are not ever directly measured it is difficult to know from this research exactly what is doing the work. Moreover, the role of already existing social ties within the neighborhood is overlooked. Perhaps it is only those who are likely to have denser relationships with their neighbors are most likely to respond to the neighbor pressure treatment.

This last point on the density of social ties needed for social norms to activate is worth some further elucidation. Most of the studies cited in this section emphasize that stronger social ties, such as personal acquaintances, friends, or family members, motivate civic engagement (either through leading by example or direct encouragement). However, Lim (2008) for example finds that strong ties are only slightly more effective than weak ties in mobilizing people. A large body of work on get-out-the-vote campaigns supports this idea by showing that having strangers show up on your door step to ask you to vote increases turnout (e.g. Gerber & Green 2000; Gerber & Green 2008). Thus, there remains an empirical question: must dense social

[4] Of the experimental studies mentioned here, only Hopper & Nielsen (1991) tackle personal versus social norm activation in surveys with their participants, but they leave out strength and density of social connections among neighbors or identification with the community as possible factors that mediate the effects of neighbors requesting participation.

ties already exist for civic participation or does one simply need to be asked by someone to participate?

Collectively, the work reviewed in this section thus far indicates that there is something about face-to-face contact, social ties, and neighborly relations that matter for engagement in civic participation through the activation of social norms, yet the relationships between these factors remain unclear. Although I do not anticipate to sort everything out in this study, I aim to shed light on the complex process by which social norms are translated into collective action at the community level.

Individual Socioeconomic Background and Participation

There is a strong consensus in the political science literature that poorer individuals participate less actively in political and civic actions than wealthier individuals. This literature, however, relies almost exclusively on studies on established Western democracies. The poor have been found to be less likely, for example, to vote or participate in protests (e.g. Barnes and Kaase et al. 1979; Verba and Nie 1972; Verba, Nie and Kim 1978; Verba et al. 1995).

A potential mechanism underlying the relationship between socioeconomic background and participation that has been discussed extensively in the literature is social capital. In his seminal study, Putnam (2000) argues that middle- and upper-class citizens in the US have historically had high levels of social capital which they accumulated through their engagement in sports clubs and other organizations. According to Putnam and others, members of higher social classes possess more formal social networks and higher levels of social trust than the lower classes and therefore, upper-class individuals are more likely to engage in community actions (Putnam 2000; Uslaner and Brown 2005; Delhey and Newton 2003). In a number of studies on social capital, people from the upper echelons of society are argued to be more successful and satisfied with their lives and therefore, trust more in others (Delhey and Newton 2003; Putnam 2000; Alesina and La Ferrara 2000). While trust bears risks for all individuals, potential risks are harder to venture by the poor (Delhey and Newton 2003: 95). Thus, the wealthy are expected to engage in civic action more than the poor.

Yet there is also some debate surrounding whether measures of social capital focus too much on formal relationships involved in institutionalized groups, overlooking more informal

networks. Some scholars have argued that the degree to which an individual is embedded in informal social networks also explains differences in levels of social trust (Delhey and Newton 2003). Florian Pichler and Claire Denise Wallace (2009) find in a study on social capital in Europe that the upper classes possess more formal social capital. However, they do not find differences between social classes when looking at what the authors call "informal social capital" that is based on the relationships with, for example, friends and neighbors rather than members of institutionalized social clubs or workplaces. Informal social capital is measured using questions on "How often do you meet with neighbors and friends" from the Eurobarometer (2004, N= 27 000).[5] As the poor are likely to have strong ties with neighbors and friends, I expect that the poor should also be more likely to engage in communal activities.

Within the field of social psychology, studies show that poor and wealthy individuals differ remarkably when it comes to their social behavior towards others (Piff, Kraus, Côté and Cheng 2010). Some studies find that poor individuals behave more prosocially towards one another compared to wealthier individuals (cf. Piff et al. 2010; Piff and Robinson 2017). In a series of dictator and trust games, Piff et al. (2010) show that participants from the lower social stratum allocate more money to their partners than their counterparts from the higher socioeconomic stratum. In a separate experiment, they attempt to manipulate the respondents' relative social class. Participants were primed to think of their social position relative to others who are at the top or bottom of the social ladder; participants of a lower-class background measured both as objective social class and relative social class act more charitably in terms of reported percentage of people's annual salary that should be donated.

Other work suggests that socioeconomically disadvantaged individuals are generally more oriented towards others (e.g. Stephens et al. 2007; Dietze and Knowles 2016). Dietze and Knowles (2016) find that lower class individuals – measured using a group-based measure of self-reported social class – are more attentive towards others than their upper-class counterparts. In visual experiments, the authors track where participants look at when walking along the street. In two separate studies, they track participants' eye movements when looking at images. These experiments find that lower class individuals spend more attention to others around them than the wealthy. Stephens, Markus and Townsend (2007) show that lower-class individuals take note of what others are doing and make choices based on what previous

[5] For an overview of different approaches to social trust see Delhey and Newton (2003).

participants have chosen; by contrast, middle class individuals make choices independent of previous participants. In a series of short experiments, the authors show that lower class participants choose pens and images similar to other participants. They also find that lower class individuals reacted positively when presented with a hypothetical scenario in which one of their friends just had bought the same car as they had.

The tendency of the poor to be more generous and other-regarding is paradoxical given that pro-social behavior is costly in terms of time, money, and/or effort (Piff et al. 2010) and lower-class individuals typically possess less resources and fewer opportunities when it comes to education and labor market participation. Compared to wealthier individuals, they are more likely to be facing hostile environments in which crime and domestic violence are not uncommon (Fahmy, Williamson and Patazis 2016; Huang, Laing and Wang 2004). Moreover, poorer individuals are more economically vulnerable than their wealthier counterparts. Whereas wealthier individuals possess higher levels of control and freedom of choice in everyday decision following their favorable economic situation, the poor have been found to mostly lack self-control (Kraus, Piff and Keltner 2009). This implies that poorer individuals should focus on the self and less on others to counter their relative disadvantage in life.

However, this is not what the literature finds. Instead, the difficulties and lack of control that the poor face leads to an orientation towards others among them, whereas security and freedom of choice lead to self-orientation among the wealthy (Robinson and Piff 2017; Kraus, Rheinschmidt and Piff 2012; Piff and Robinson 2017). The experienced uncertainty in their everyday life combined with a lack of individual control leads to an "external, other-oriented focus" of the poor (Piff and Robinson 2017: 6). In other words, as poorer individuals are more economically vulnerable, they are also more vulnerable to others (Lachmann and Weaver 1998; Kraus, Rheinschmidt and Piff 2012; Kraus, Piff and Keltner 2009). Thus, poorer individuals respond to experiences of dismissive and sometimes hostile environments with higher levels of prosocial behavior compared to wealthier individuals (Piff, Kraus, Côté and Cheng 2010). Robinson and Piff (2017) understand pro-sociality among lower class individuals as "adaptive responses to reduced personal control and uncertainty" by which the individuals minimize risks they are facing in a hostile environment.

Sociologists have put forward the argument that people from a similar socioeconomic background are considerably more likely to socially interact with each other (for a research

overview see McPherson et al. 2001). This tendency to interact with others who share similar characteristics – that is referred to as social homophily in the literature – should also affect how people behave politically. The idea is that people might be more strongly influenced by similar others.

In turn, one might expect neighbors from a lower socioeconomic background to be generally more likely to socially bond with each other and to build out dense social ties. Some of the sociological literature further shows that less affluent individuals tend to be more oriented towards their immediate local environment than their more affluent counterparts (van Eijk 2010; Pinkster 2007). As Bridge (2002: 12) suggests, "neighborhood relations might be relatively more significant for those with limited economic resources and mobility" and thus, affect poorer individuals to a larger extent than wealthier individuals.

Social Context and Participation

Unlike the importance of individual characteristics such as socioeconomic background on participation, the effects of community composition along class lines has received less scholarly attention. Instead of asking how individual socioeconomic status affects collective action at the local level, this line of work considers how the socioeconomic context an individual lives in affects his or her likelihood of participation. The Columbia school in political participation research emphasizes that individuals do not act in isolation from their social environment but are affected by the social structure in which they are embedded (e.g., Lazarsfeld et al. 1944; Berelson et al. 1954). Following this tradition, Huckfeldt (1979), Giles and Dantico (1982), Kenny (1992) and others have analyzed the effects of social neighborhood context on civic and political participation. Huckfeldt (1979) uses survey data from Buffalo, New York to examine the effects of high versus low socioeconomic status neighborhoods on individual versus social modes of political participation.[6] He shows that living in higher status social contexts – measured in the percentage of higher status individuals living in this neighborhood – increases the levels of participation of higher-class citizens. At the same time,

[6] Huckfeldt (1979: 585) differentiates between individually based participation and socially based participation. The latter includes participation in a political party between elections as well as at election time, giving money to a party or candidate, working to get people registered to vote, take an active part in political campaign, join groups working to improve community life, inform others in my community about politics, join and support a political party. According to Huckfeldt, all these actions are based on social interaction with other participants or political organizations. Individually based forms of participation include, for example, voting in an election or sending a protest message to a political leader (ibid.).

it decreases the participation of lower-class individuals in these neighborhoods. Yet, Huckfeldt also shows that lower-class individuals are more likely to participate when living in lower status contexts. In an early study from 1937, Tingsten finds that individuals from the working class are more likely to vote when living in working class neighborhoods in Stockholm compared to workers living in other neighborhoods. More recent work by Andersen and Health (2002) and Andersen et al. (2006) similarly finds that the effects of social context on voting are most pronounced for individuals of a working-class background in the United Kingdom.

More recently, scholars like Eubank et al. (2018), Siegel (2009), Rolfe (2012) and McClurg (2003) have focused on social networks and the influence of peers, family and elites on the political and civic behavior of an individual. Other than the notion of social neighborhood context which refers to the social structure or socioeconomic composition of a neighborhood, social networks describe the communication structures and social connections within the neighborhood (Huckfeldt and Sprague 1987; Huckfeldt 2007). Focusing on social networks of Ugandan villages, Eubank et al. (2018) show that the links between peers are crucial for the participation in less salient local elections where media reporting is relatively rare. Thus, social networks can make up for lack of information in spurring participation. Others, like Pavlova and Silbereisen (2015), observe in the case of East Germany that in contexts in which the participants reported that their community at large supports their needs and in which they felt a greater sense of community political and civic participation of citizens was increased.

In line with this previous literature, I expect the effects of social norm activation to be strongest in areas that are homogeneous, and specifically that poor individuals are particularly more likely to participate in collective action that benefit the community when living in homogeneously poor communities versus homogeneously wealthy or mixed communities. Although the wealthy may possess high levels of social capital more broadly speaking, I do not expect them to derive these connections from their neighbors, but rather their wider networks developed through business, country clubs, and other groups that are not necessarily located in close proximity to them.

Theoretical Model: Social Norms and Civic Engagement Across Socioeconomic Groups and Contexts

The idea that people are, at least in part, driven to action by what those around them are likely to do and to expect of others should not be surprising. Yet, as Schultz et al. (1995, 114) underscore, many studies on community collective action do not seriously consider characteristics of the communities they are working within. With this study I can attempt to better understand to what extent to which social norms are associated with community mobilization across different types of individuals, on the one hand, and varying community contexts, on the other. These outcomes are important for understanding how to motivate different types of people to engage in civic initiatives. If different factors are relevant in varying ways for different types of people (e.g., the poor versus the wealthy) when it comes to civic behavior, then perhaps different strategies for mobilization are needed in different contexts.

This review of the literature points to a number of potential moderators between social norms and behavioral outcomes, which I propose to measure at the individual and community level, including: 1) Social monitoring or sanctioning, 2) solidarity and social belonging and 3) social trust and 4) social obligations to reciprocicate.[7] Thus, in this study, I aim to assess how social norms are translated into community engagement. As the local environment is typically the level of aggregation which affects people most directly in their everyday lifes, I also expect social pressure to be localized. And if social pressure is localized, we may expect solidarity, monitoring, social turst and reciprocity to play a role in certain local contexts but not in others. I suggest that when social ties among neighbors are denser, this should moderate the effect of social pressure on the civic engagement of the citizens. The model is presented in figure 1.

[7] I note that these factors may be highly correlated with one another (e.g., when solidarity among community members is high, they may also be more likely to trust each other), but given that so many studies consider these mechanisms separately or lump then all together as "social capital", I believe that it is worth investigating how these potential moderators work either in tandem or perhaps outweigh one another in the relationship between social norms and actions. In addition to these factors, some scholars argue that it is only when generalized social norms become internalized and transform into intrinsic personal norms that behavioral outcomes result (Schwartz 1970 & 1977; Hopper and Nielsen 1991; Nigbur et al. 2010). While this distinction is theoretically intuitive, in practice, sorting out whether social norms work through personal norms or vice versa is complicated and beyond the scope of the project presented here.

Figure 1: The Translation of Social Norms into Civic Participation

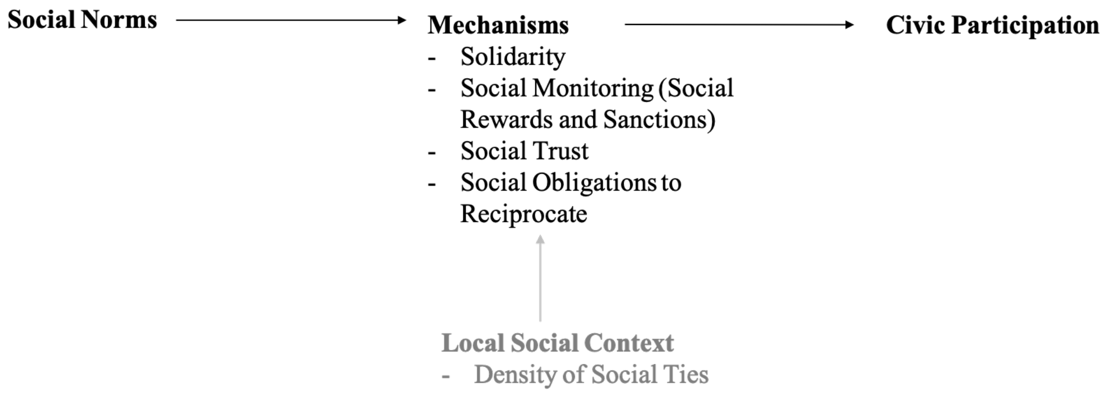

Moreover, I expect the impact of these mechanisms to vary by the socioeconomic background of the individuals. This effect should be moderated by two contextual variables, namely the socioeconomic composition of the neihgborhood and the density of social ties. As previous research has suggested, poor individuals are more dependent on their neighbors to deal with everyday problems and, in some cases, to secure their livelihoods. In turn, they should be more likely to participate in community actions as a result of social monitoring, a sense of community belonging and social obligations to reciprocate. We may also expect them to be more likely to trust their neighbors, though not necessarily also to have higher levels of generalized social trust. Moreover, the density of social ties and the socioeconomic composition are potential moderators increasing the effect of these mechanisms. When living in socially dense communities or homogeneously poor communities, social monitoring and belonging or the obligation to help each other should be higher than in communities that are less dense and those that are socioeconomically mixed. I propose the following model to capture these effects (see figure 2).

Figure 2: Social Context, Norms and Civic Participation Among the Poor

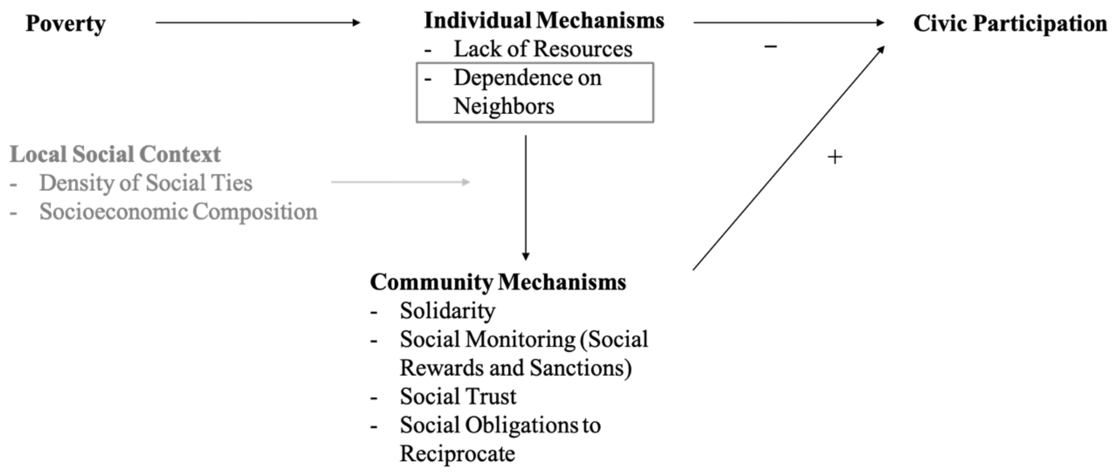

In contrast to much of the extant work, this study also proposes to capture previous engagements among neighbors as well as relationships and expectations amongst them. To truly understand civic engagement among neighbors in particular, we must consider their past interactions and personal connections with one another. Participants are not blank canvasses as they walk into an experiment but much of behavior is conditioned by previous personal experiences. Norms of reciprocity, obligation, interpersonal trust, fear of monitoring/sanctioning, and group belonging all rely on the notion that neighbors have built up histories of interactions with one another. Previous work by Ostrom and Walker (2003) suggests that cooperation requires pre-existing social relations over time. In line with these suggestions, in behavioral games, Schaub et al. (2020) find that the people tend to trust less in poor indivdiuals who they did not know – and even less if both cooperation partners were poor. In the experiment, cooperation partners were allocated by the researcher and the participants did not know each other.

Again, these findings undergrid an interest in neighborly behavior—a setting in which people have increased likelihood of prior engagement with one another—rather than just social behavior and norms more broadly. I formulate the hypotheses below that address how types of relationships may vary across different types of communities.

Hypotheses

As noted, my theoretical framework is based on some of the earlier literature in political participation and research on civic engagement, but also draws on findings from social psychology and sociology on the social behavior of poor and wealthy individuals and the role of social norms and obligations within poor versus wealthy communities. By holding the clean-ups in a place that is, on average, equidistant to our participants of varying socioeconomic backgrounds, the experimental design allows me to hold constant the costs of civic participation including effort, material cost, and time. Due to the fact that we cannot randomly assign socioeconomic status or neighborhood composition to individuals, some of our hypotheses are evaluated through observational analyses. Yet, I do experimentally manipulate what I expect to drive our outcomes: the activation of a social norm and moderators expected to translate that norm into behavior by sending neighbors to recruit participants in a community clean-up initiative. Social norms are not uniformly ever-present; it is only when they are activated and are relevant that they take effect (Cialdini et al. 1990, 1015).

First, looking at the individual level, I expect that neighbor community organizers will be more successful at recruitment. Following Neilsen and Hopper (1991, 202), I posit that neighborhood block leaders can activate social norms surrounding community clean-ups.

H1a: On average, neighbor recruiters will successfully recruit more participants to the clean-up events than community outsiders. I measure this by presence at the clean-up event first among the sample of initial HoH recruiters, and second, among their subsequent recruits.

H1b: Respondents will be more likely to participate when they have been recruited by a neighbor than by a community outsider. I measure this by presence at the clean-up event first among the sample of initial HoH recruiters, and second, among their subsequent recruits.

I also include an alternative dependent variables measuring the degree of engagement in the clean-up by counting the number of trash bags filled and by noting down whether they wear buttons that label them as community mobilizers during the event (among initial recruits only)
.

H1c: Respondents will collect more trash/ be more likely to wear a button that labels them as "community mobilizers" during the clean-ups when they have been recruited by a neighbor than by a community outsider.

Moreover, I expect that neighbor versus outsider recruiters will activate higher scores on the community mechanisms that I have identified to translate social norms into civic engagement: 1) community sanctioning (e.g., Sinclair 2012) and 2) generalized social trust (e.g., Putnam 2005); 3) solidarity, or group attachment (Baldassarri 2015), and 4) reciprocity (Cialdini and Goldstein 2004; Gouldner 1960).

H2a: In a post-treatment survey, I expect those who were recruited by neighbors to have higher individual scores on community mechanisms than those recruited by community outsiders (those not at the event will be called and asked to complete a follow-up survey on why they did not participate). This effect is expected to hold across both the sample of initial HoH recruiters and their subsequent recruits. Note that these are observational analyses.

H2b: I expect high scores on community mechanisms to increase the effect of neighbor recruitment on participation and the number of trash bags filled. I will use causal mediation analyses as described by Imai and Yamamoto (2013) to test if the proposed mechanisms drive the relationship between neighborly recruitment and participation.

I hypothesize that the moderators of social norm translation into civic action may vary across poorer and wealthier individuals. In particular, following previous studies cited above, I hypothesize that poor neighbors should in general be more likely to build out denser ties with one another and have higher expectations of obligation and reciprocity from one another than wealthier neighbors (e.g. Piff 2010). However, there is reason to believe they may be less likely to have generalized social trust (e.g., Putnam 2000) but higher trust in neighbors.

H3a: Poorer individuals will be more likely to fear community sanctions and to be monitored by their neighbors, to have stronger feelings of obligation, and a stronger sense of duty to reciprocate with their neighbors than wealthier individuals with theirs. Wealthier individuals will have more generalized social trust than the poor but the poor will be more trusting in their neighbors than the wealthy. Note that these are observational analyses.

I also expect the effect of neighbor recruitment versus recruitment by an outsider to be stronger for the poor compared to the wealthy.

H3b: Assuming H3a is true, on average, poor participants will both be more likely to participate themselves and will be more likely to successfully recruit their neighbors for participation in the clean-up event and fill more trash bags/ wear the buttons compared to wealthier individuals when asked by a co-local rather than community outsiders to participate in the clean-ups.

As noted, I expect that poor individuals should be more socially-oriented and should build out denser social ties when living in neighborhoods in which many others share their social status. Thus, based on this previous research, I derive the following hypotheses regarding the role of social ties and the socioeconomic composition of the neighborhoods:

H4a: Community mechanisms, with the exception of generalized trust, are stronger among members within the homogeneously poor neighborhood than the socioeconomically mixed neighborhood. (Observational analysis).

H4b: Participation in community initiatives as measured by participation among initial recruits (community mobilizers) for participation, will be higher among individuals living in homogeneously poor neighborhoods than those living in socioeconomically mixed ones. (Observational analysis).

H4c: Recruitment by neighbor mobilizers will drive higher participation in community initiatives and a stronger engagement (i.e. the number of trash bags filled, wearing a button or not) in homogeneously poor neighborhoods than in socioeconomically mixed (examined among initial recruits and by looking at the number of additional recruits by community mobilizer).

I formulate the final hypotheses on the effect of social context on the participation of poor individuals more specifically. I test whether citizens ability to mobilize their neighbors to participate in a community clean-up event is associated with the socioeconomic composition of the neighborhood. The following analyses will be observational:

H5a: Poor individuals specifically are more likely to participate in civic action when living in communities in which they constitute the socioeconomic majority than poorer individuals living in socioeconomically mixed neighborhoods (among initial recruits).

H5b: Poor individuals specifically will be more successful in recruiting their neighbors when living in neighborhoods in which they constitute the socioeconomic majority than poorer individuals living in socioeconomically mixed neighborhoods (number of additional recruits by community mobilizer).

Thus, with this research design, I will attempt to gain purchase on the separate and potentially differing effects of socioeconomic status, neighborhood composition, and density of social ties to examine what drives outcomes in terms of participation in community initiatives. The research design is structured in a way so we can understand a myriad of differing factors that may or may not be associated with civil mobilization at the local level in Tunisia.

Research Design

In order to test our hypotheses, I propose to run a mixed-method study involving both observational and experimental data. I employ pre- and post-intervention surveys and a field experiment in two different neighborhoods in Tunisia in which I use participation in a neighborhood clean-up campaign as our behavioral outcome. I purposively choose one homogeneously poor neighborhood in which poorer respondents (lower-middle and lower class) constitute the socioeconomic majority, one homogeneously wealthy neighborhood and one socioeconomically mixed neighborhood. Yet, we draw our participants from four samples of 400 participants each: 1) poorer respondents in the homogeneously poor neighborhood; 2) poorer respondents in the mixed neighborhood; 3) wealthier/middle class respondents in the homogeneously wealthy neighborhood, 4) wealthier/middle class respondents in the socioeconomically mixed neighborhood. I am limited to purposively selected just three neighborhoods for this study by funding, but such a study could serve as a pilot for a larger and more comprehensive sample should the findings warrant such scaling up of the research. All neighborhoods will be located in the same governorate (Tunis) and within a reasonable geographic distance to one another.

The study mimics Sinclair's (2012) design of sending local versus non-local recruiters to households in a non-Western setting, and adds an additional component whereby each initial recruit will then be asked to recruit more neighbors to join a community clean-up event (as opposed to voting in her study). Yet, while Sinclair is able to compare indivdiuals who were not visited by a local or non-local canvasser to those you were informed about the upcoming elections, I compare indivdiuals who were recruited by locals to those who were asked by non-local recruiters to join. Using this method, I seek to observe the process by which social norms translate into collective action at the local level through measuring perceptions of potential moderators across our experimental arms, community members of differing backgrounds, as well as communities of differing socio-economic compositions. On average, I expect it is only when a neighbor asks for participation in a community initiative that the social norm of keeping one's community clean is strongly activated and the community mechanisms suggested in my model are expected to translate this norm into civic action. The survey provides various measures of our potential moderators in this process.

Case Justification

I choose the participation in neighborhood clean-up campaigns for several reasons. First, together with my local partners, ELKA and Zero Waste Tunisia, I am actually able to organize clean-up events in different neighborhoods and to ask people to join. By organizing actual clean-up events and asking people to participate, we will be able to use a hard measure for community participation. Most existing studies have relied on more indirect measures using survey questions in which respondents are asked to state their preferences (e.g. How likely would you be to participate? Answers: Very likely, somewhat likely, somewhat unlikely, not likely at all). There are concerns that such outcomes suffer from social desirability bias. In this study, I can compare between these two outcomes of reported likelihood of participation and actual participation rates across individuals and communities. Moreover, since I study actual behaviors surrounding community clean-up initiatives, which will provide a benefit to the communities included in our study regardless of our scholarly findings. By combining a field experiment with pre- and post-intervention surveys, I will dig into whether people participate when social norms of engagement are activated on the community level and if so, why individual what translates these norms into action. Moreover, I will be able to investigate whether compliance with neighbors versus strangers varies between different socioeconomic groups and social contexts.

I select Tunisia, a lower middle income[8] country in the Middle East, because much like other developing democracies, the country faces severe waste management problems (cf. Boh 2016; Abdulrahman 2018; Foroudi 2019). For example, in Tunis, between 0.58 and 0.82 kg of solid waste are produced per person per day [mean value of 0.65 kg] (Aydi et al. 2013). The municipalities are responsible for the trash collection and citizens are asked to put their waste in plastic bags close to the nearest road where they will be picked-up collectively. Around 80 percent of the municipal solid waste (MSW) gets collected in the big cities, yet 20 percent of MSW ends up, for example, on the streets and beaches (Boh 2016). As we will show later in this paper, waste management problems equally affect neighborhoods of differing socioeconomic composition in Tunis.

Tunis also makes for a good case study because an important precondition for community initiatives to become successful is the general awareness of the problem that these initiatives are targeting. Tunisians are very aware of the problem of trash in public spaces. In the 2018 wave of the Arab Barometer, 77 percent of the Tunisians in the survey responded that trash represents a very serious problem, followed by 18 percent who think that it represents a somewhat serious problem. After splitting the sample into two different income groups - below and above median income - 75 percent of the respondents with an income below average responded that it represents a very serious problem, which is comparable to 80 percent of the respondents with an income above average. As such, we expect that we will not need to convince our recruits that the neighborly clean-up initiative is a good thing, representing a social norm, but we will need to persuade them to expend the time and effort to join it and actually act on this norm.[9] As Schwartz (1977) points out, while most people will agree with an outcome associated with a social norm, not everyone will be willing to comply with this norm when it comes to actual behavior. While many people may internalize social norms and believe in them, they may still not act in accordance with them (Hopper and Nielsen 1991). Thus, clean-up initiatives in Tunisia allow me to examine the process of how social norms translate into behavior and to test the mechanisms underlying this relationship.

[8] According to the World Bank: https://data.worldbank.org/?locations=XN-TN.

[9] We will, however, check that this holds across the wealthy and the poor in our sample. It could be that attribution of responsibility for clean-ups of public spaces varies across the poor and the wealthy such that some see it as their civic duty whereas others see it as the responsibility of the municipality or higher-level government.

Such cleanups are not unheard of in Tunisia. In some municipalities for instance, citizens have organized clean-up campaigns themselves, for example, after sit-ins and strikes and after the recent presidential elections in October 2019 (Tunisie numérique 2012; Chennaoui 2019). In Tunis, waste pickers, so-called *berbecha*, collect plastic waste from the streets and sell it to middlemen recyclers (Foroudi 2019). Thus, given the importance of the problem and the seemingly awareness of it among the Tunisian citizens of differing socioeconomic backgrounds, I think that organizing a waste collection initiative will attract Tunisians from different social classes and help to eliminate pre-experimental biases.

Finally, community participation has been discussed among practitioners and scholars as a potential tool to improve waste management on the local level, to protect the environment and to decrease risks of diseases (e.g., Atienza 2008; Abeyewickreme et al. 2012). Solid waste management is becoming an increasingly severe problem for municipalities not only in Tunisia but all around the world and, in particular, in most developing countries where population rates are on the rise and national economies are expanding. Higher spending capacities of an increasing number of citizens as well as continuing urbanization lead to higher waste production (Guerrero et al. 2013; Minghua et al. 2009; Atienza 2008). At the same time as the total amount of trash produced increases steadily, these countries face financial as well as institutional difficulties - including a lack of political will - to deal with the problem (Atienza 2008; Guerrero et al. 2013). Typically, municipalities are responsible for the collection of the solid waste such as plastic and food waste and any other types of garbage. However, most municipalities manage to collect only a certain percentage of the actual waste, while the rest ends up on the street, in parks and lakes. Therefore, community participation is seen as becoming an increasingly important tool to help reducing the pollution of the environment.

Methods

In field experiments, individual behavior is measured under conditions that are similar to a real-life situation. Moreover, instead of asking about the stated preference of each respondent as in survey experiments, I will measure real behavior – participation in a clean-up campaign. As part of the experiment, we organize cleaning campaigns within three neighborhoods that will actually take place and thus, I do not ask about hypothetical situations. Participants in this study will be asked by our enumerators to come together on a Sunday morning to collect the trash in an area in need of clean-up. The enumerators will also ask the participants to recruit

more contacts from their neighborhood to joint them, allowing me to examine who is more successful in recruiting others from the neighborhood to join the event.

I conducted a first research trip to Tunisia in November 2019 to collect information on neighborhoods in Tunis for inclusion in this study. Regarding our clean-up sites, I searched specifically for areas that are of roughly equal importance to potential participants with the ideal setting being some natural area (a marsh, lake, or other natural setting) that all can agree is in need of clean-up and that all have equal access and are about equi-distant to get to. I collaborated closely with the National Statistics Institute in Tunis which provided data from the INS census 2014 – including socioeconomic indicators on all neighborhoods in Tunis. For the field experiment, I chose three neighborhoods that are located in Le Kram, La Marsa and La Goulette – three neighboring municipalities within the Governorate of Tunis. Le Kram West as homogenously poor, La Marsa Corniche as homogeneously wealhty and La Goulette Casino as socioeconomically mixed neighborhood are located near the beach. Therefore, I chose the beach as our clean-up side for the experiment. We will also run a pilot study two weeks before we plan to start recruiting for the first clean-up event. The pilot study will be conducted in Hammam-Lif which is in the Governorate of Ben Arous and has a beach where we can organize the pilot study and a small clean-up event with 50 recruited HoHs.

Recruitment of Participants

I will hire local enumerators[10] from a local enumerator firm (ELKA Consulting in Tunis) who will recruit four groups of 400 randomly selected individuals in three neighborhoods in Tunis (one individual per household).[11] The four samples comprise of 400 individuals each as follows: 1) a sample of poor individuals living in a homogenously poor neighborhood (Le Kram West); 2) a sample of poor individuals living in a socioeconomically mixed neighborhood (La Goulette - Casino); and 3) a sample of upper middle class to wealthy indivdiuals in a wealthy neighborhood (La Marsa – Corniche), 4) a sample of upper middle

[10] ELKA has a big pool of enumerators from which we will be able to choose enumerators who share similar characteristics such as religious and socioeconomic background etc. This will allow us to minimize enumerator effects. We could also select enumerators with a lower middle class background to recruit individuals in the poor neighborhood and with a upper middle class background to recruit individuals in the wealthy neighborhood.

[11] We will give out printed maps with the exact neighborhood boundaries to the enumerators. Enumerators will be asked to only include households within the boundaries of the three neighborhoods that have been selected for this study.

class to wealthy individuals living in a socioeconomically mixed neighborhood (La Goulette - Casino). [12] Half of the enumerators who will invite the participants to join the clean-up events will be hired from within the neighborhood and the other half from another neighborhood in Tunis. All enumerators are still Tunisian, so the randomized variation remains simply one of local proximity of inhabitance. We will experimentally test whether this affects likelihood of participation in the clean-up event and how this varies across different individuals, neighborhoods, and community mechanisms.

When visiting a household, the enumerator will gain informed consent for participation in the study from the head of the household (HoH) (or next eldest member of the household). Each HoH will be asked to participate in a small survey (approx.. 15 min) about social demographics of the person and the household, social ties within the neighborhood, and whether the person has previously participated in community events. All surveys will be conducted by enumerators who do not live in the same neighborhood. Only after conducting the first survey, a second enumerator will join announcing whether they are from the neighborhood or not, and invite the HoH to join the neighborhood clean-up. They will also provide flyers with information about the event (description of the event, date, meeting place) to each HoH for sharing with neighbors. The HoHs will be actively encouraged to recruit up additional participants outside of his/her family but living within his/her neighborhood (the neighborhood will be defined by a map on the flyers) to attend the clean-up event.[13] The enumerators will also give a button to each HoH to wear at the event which labels them as “community mobilizers”. Moreover, the enumerators will inform the respondent that they will need to register as a team in the beginning of the clean-up (see recruiter protocol). The idea here is to see variation in the ability to recruit neighbors across the samples and whether enumerator status as a community insider versus outsider affects the propensity to attend as well as recruit for the clean-up events. Finally, the enumerators will ask for phone numbers to follow-up with only these initial recruits who do not show up at the clean-up event, once the event is over simply to ask why they did not show up to the event. We will also use these numbers to send text message reminder of the event to encourage participation. Phone numbers will only be collected after receiving informed consent and will be deleted by the enumerators after the follow-up one week after the event. The researchers will not get access to these phone numbers.

[12] The research leads on this project have received data on socioeconomic variation between different neighborhoods in Tunis based from the 2014 census on which the sampling design can be based.
[13] Participation/ recruitment will be restricted to people who live in the neighborhood.

Lottery prizes will be announced beforehand as being inexpensive gift cards for phone credit, for example. We will announce winners at the event. Participants will also be provided with water and trash bags. In the face of the Covid-19 pandemic, we will also provide latex gloves and hand sanitizers at the event. All participants will be asked to keep a distance of 1.5 meters to the other participants. Participants will be sent out in groups of three people maximum to clean up the beach together. We are in regular contact with our local partner to stay informed about the current developments with regards to Covid-19 in Tunisia.[14]

Flyers provided by neighbor recruiters will have additional information that says "Come and join your neighbors!" while flyers provided by community outsiders will not have this additional information. The buttons will say "I am a community mobilizer!" for the treatment and the control group. During the event, we will ask all recruitees and mobilizers to come together so we can note down the number of successfully recruited neighbors per mobilizer. Our surveys at the clean-up event and follow-up surveys with those who did not show up (described in further detail below) will also allow us to track recruitment and participation patterns. If a recruitee received more than one flyer or was recruited by multiple persons, we will ask about this in the survey and control for it in our analyses.[15]

The Event: Cleaning the Neighborhood

The actual clean-up events will be organized on a Sunday about a week after initial recruitment of HoHs takes place. We anticipate three such events at the beaches in Le Kram, La Marsa and La Goulette, being held simultaneously. At the events, enumerators will check in each head of household and his/her recruits. They will run another small survey (about 15 minutes) about socioeconomic status and previous engagement in community events with all new recruits (those the heads of households recruited) to the events. Enumerators will also run a second

[14] We are very aware of the risks connected to conducting this research in times of a pandemic. We have already postponed the fielding of this experiment twice due to the uncertainty surrounding the Covid-19 virus. The project will only be fielded in November if the situation allows it. Local enumerator firms resumed work and started running face-to-face interviews again given the overall low numbers of Covid-19 cases in Tunisia. Also, several local initiatives, for example, to clean-up the beach in La Marsa and the streets in La Soukra (with more than 50 participants) were organized in August. For information see https://www.facebook.com/TounesCleanUp (link to the official Facebook page of CleanUp Tounes).

[15] We will run analyses coding this case as a successful recruit for each community organizer named by the recruitee, and dropping these cases out of the dataset to check for robustness of our findings.

survey (about 10 minutes) with our HoHs to make sure the connections between them and their recruits are understood, their recruitment techniques, as well as their reasons for showing up.

At the end of each clean-up, we would like to organize a social event to bring together the participants. At the event, my local partner organization Zero Waste Tunisia[16] will provide some information on their work which aims to sensibilize people to keep public spaces clean and to reduce plastic waste. Zero Waste Tunisia will also provide information on brand audits, an initiative that aims to collect the names of the brands on the waste that was collected, and train people to join this effort if they are interested. Engaging with the team of Zero Waste Tunisia and joining the brand audit will be voluntary for all participants and is aimed at trying to get members of the crowd to engage with one another and the general issue of waste pollution.

ELKA will also create Facebook groups for each neighborhood to see how many participants sign up for the Facebook groups and whether people continue to organize clean-ups in the future or exchange information.

Finally, we will run a phone survey (approx.. 5 minutes) with those HoHs who have decided not to join the events.

Measurement, Neighborhood Selection and Hypotheses Testing

I will use different outcomes in the analysis to test our hypotheses. First, we use a binary outcome measuring whether the initially recruited HoH (N=1600) joins the clean-up event or not. This allows me to compare the participation rates among the three samples. We will also be able to test whether people who received the neighborly recruitment treatment were more likely to join the experiment than those who were recruited by a community outsider and how this effect differs among the different socioeconomic groups and neighborhoods (please find our SES measurements in the appendix).

At the secondary recruitment level, where HoHs recruit other participants, I also capture the number of people recruited by a community mobilizer as measure of recruitment, and the

[16] Please find more information on Zero Waste Tunisia on Facebook: https://www.facebook.com/zerowastetunisia/.

proportion of people each initially recruited HoH was able to subsequently recruit for the clean-up event as a measure of successful recruitment. Finally, I use the overall participation rate (participants by number of inhabitants) per neighborhood as an outcome in the analysis.

Morevoer, I use the number of trash bags filled during the event and whether the HoH wears the button that was provided will be used as alternative outcome measures. Finally, we use the number of people who have signed up for the facebook group in each neighborhood as an community level outcome measure.

In order to measure community mechanisms of social engagement, I will include a series of questions in the recruitment survey about these (please see the appendix for more details). We also divide our sample by types of respondents according to various measures of socio-economic status, and by types of social contexts in which they live. A mix of t-tests and regression analyses (OLS and logistic regression) and mediation analysis will be employed to test the hypotheses.

Table 2. Research Questions, IVs, and DVs

Hypothesis	Independent Variable	Dependent variable
H1a: On average, neighbor recruiters will successfully recruit more participants to the clean-up events than community outsiders. I measure this by presence at the clean-up event first among the sample of initial HoH recruiters, and second, among their subsequent recruits.	Mode of Recruitment (neighbor vs. stranger)	Number of sucessufully recruited neighbors (among HoHs and their recruitees)
H1b: Respondents will be more likely to participate when they have been recruited by a neighbor than by a community outsider. I measure this by presence at the clean-up event first among the sample of initial HoH recruiters, and second, among their subsequent recruits. I will measure this by presence at the clean-up event first among the sample of initial HoH recruiters, and second, among their subsequent recruits.	Mode of Recruitment (neighbor vs. stranger)	Participation (coded as 0 “no” 1 “yes”) among HoHs first, and second, among their recruitees.

H1c: Respondents will collect more trash/ be more likely to wear the button during the clean-ups when they have been recruited by a neighbor than by a community outsider.	Mode of Recruitment (neighbor vs. stranger)	Number of trash bags collected (among participants), wearing a button as "community mobilizer"
H2a: In a post-treatment survey, we expect those who were recruited by neighbors to have higher individual scores on community mechanisms than those recruited by community outsiders (those not at the event will be called and asked to complete a follow-up survey on why they did not participate). This effect is expected to hold across both the sample of initial HoH recruiters and their subsequent recruits.	Mechanisms:<br>(1) the density or strength of social ties; 2) feelings of obligations or the duty to reciprocate; 3) generalized social trust; 4) community monitoring and sanctioning; and 5) local group identification, solidarity, or belonging)	Participation among HoHs first, and second, among their recruitees.
H2b: I expect high scores on community mechanisms to increase the effect of neighbor recruitment on participation and the number of trash bags filled. I will use causal mediation analyses as described by Imai and Yamamoto (2013) to test if the proposed mechanisms drive the relationship between neighborly recruitment and participation.	Mechanisms X Mode of Recruitment	Participation among HoHs first, and second, among their recruitees. Number of trash bags filled (among participants only)
H3a: Poorer individuals will be more likely to fear community sanctions and to be monitored by their neighbors, to have stronger feelings of obligation, and a stronger sense of duty to reciprocate with their neighbors than wealthier individuals with theirs. Wealthier individuals will have more generalized social trust than the poor but the poor will be more trusting in their neighbors than the wealthy. Note that these are observational analyses.	SES	Community Mechanisms
H3b: Assuming H3a is true, on average, poor participants will both be more likely to participate themselves and will be more likely to successfully recruit their neighbors for participation in the clean-up event and fill more trash	SES X Mode of Recruitment	Difference in participation for neighbor recruiters and outsider recruiters, among HoHs first, and second, among their recruitees. Number of trash bags filled.

bags/ wear the buttons compared to wealthier individuals when asked by a co-local rather than community outsiders to participate in the clean-ups.		Wearing button as community mobilizer.
H4a: Community mechanisms, with the exception of generalized trust, are stronger among members within the homogeneously poor neighborhood than the socioeconomically mixed neighborhood. (Observational analysis).	Neighborhood Social Context (homogenously poor vs. mixed)	Social Norm Moderators with the exception of generalized social trust
H4b: Participation in community initiatives as measured by participation among initial recruits (community mobilizers) and the participation rate/ the number of people who signed up for the facebook group will be higher among individuals living in homogeneously poor neighborhoods than those living in socioeconomically mixed ones. (Observational analysis).	Neighborhood Social Context (homogenously poor vs. mixed)	Participation (HoHs only), participation in facebook group, overall participation rate
H4c: Recruitment by neighbor mobilizers will drive higher participation in community initiatives and a stronger engagement (i.e. the number of trash bags filled, wearing a button or not) in homogeneously poor neighborhoods than in socioeconomically mixed (examined among initial recruits and by looking at the number of additional recruits by community mobilizer).	Interaction between Neighborhood Social Context (homogenously poor vs. mixed) and mode of recruitment (neighbor vs. stranger)	Participation (among HoHs and their recruitees). Number of trash bags filled. Wearing button as community mobilizer.
H5a: Poorer individuals specifically are more likely to participate in civic action when living in communities in which they constitute the socioeconomic majority than poorer individuals living in socioeconomically mixed neighborhoods (among initial recruits). (Observational analyses).	*Sample of Poor Individuals:* Neighborhood Social Context (homogenously poor vs. mixed)	Participation (among HoHs only)
H5b: Poorer individuals specifically will be more successful	*Sample of Poor Individuals:*	Participation (among secondary recruits only)

in recruiting their neighbors when living in neighborhoods in which they constitute the socioeconomic majority than poorer individuals living in socioeconomically mixed neighborhoods (number of additional recruits by community mobilizer). (Observational analyses).	Neighborhood Social Context	

Conclusion

In this research proposal, I focus on drivers of individual participation in collective action at the local level, contributing to the literature on collective action among different social classes and the role of social context versus individual attributes in community participation. This approach allows me to unpack what drives community participation. To the best of my knowledge, this proposal introduces a novel approach to the study of social norms and community engagement by organizing actual clean-up events for which community mobilizers will be asked to recruit others in their neighborhood and combining surveys with a field experiment on this topic.

The questions about community mobilization that I tackle in this research project are not only interesting from a research perspective, but have substantive impacts as well. My findings will provide insights into which social contexts vulnerable individuals participate in collective action and what can help to improve citizen engagement, offering important contributions to environmental policymaking for decisionmakers and practitioners in the Global South. The relationships between social norms, neighborly relations, and civic engagement is not clear in the social capital model. Does the creation of opportunities for civic engagement lead to increased social ties and therefore social engagement, which would indicate that all that is needed is for community-based activities to be initiated? Or do social ties between community members need to be activated first for community collective action to succeed, in which case community member buy-in to activities must first be generated? And finally, does the success of community initiatives depend on the existing stock of social capital in a community? This would suggest that policymakers should focus first on the strengthening of such stock potentially through the altering of educational curricula, refocusing urban planning on the creation of neighborhood environments that bring neighbors together in parks or on sidewalks

or in museums, and labor laws that allow family relations to remain strong. Our work contributes to understanding which of these approaches is most needed.

I expect that the community can effectively activate social norms needed for community participation. Issues of environmental protection are often addressed in a top-down manner on the national level. Yet, if my theoretical expectations are correct, the active engagement of locals is essential to achieve environmentally friendly behavior among community members. Strategies to increase community engagement would need to be tailored to fit varying community contexts and social processes that may differ across types of individuals. Additional research is needed that puts the focus more on the municipality or local level at which trash collection is organized. Future studies should further pay attention to how possibilities for increased citizen engagement can be opened up and also be supported in the long run. Therefore, this research project can make an important contribution to the study of importance of local governance and development more broadly.

REFERENCES

Abdulrahman, Abdulmumin (2018): Solid Waste Management in Tunisia, EcoMENA, published on June 2, 2018, online available: https://www.ecomena.org/solid-waste-management-tunisia/, [15.09.2019].

Abeyewickreme, Wimaladharma/ Wickremasinghe, A.R./ Karunatilake, Kankanige/ Sommerfeld, Johannes/ Kroeger, Axel. (2012): Community mobilization and household level waste management for dengue vector control in Gampaha district of Sri Lanka; an intervention study. Pathog Glob Health. 2012, 106(8), 479-87.

Adler, Nancy E./ Epel, Elissa S./ Castellazzo, Grace/ Ickovics, Jeannette R. (2000): Relationship of subjective and objective social status with psychological and physiological functioning: Preliminary data in healthy white women, Health Psychology, 19, 586–592.

Alesina, Alberto, and Elesina La Ferrara (2002): Who trusts others? Journal of Public Economics 85 (2):207-234.

Atienza, Vella A. (2008): A Breakthrough in Solid Waste Management: through Participation and Community Mobilization: The Experience of Los Banos, Laguna, Philippines, retrieved from: http://www.apu.ac.jp/rcaps/uploads/fckeditor/publications/journal/RJAPS_V24_Atienza.pdf, [15.10.2019].

Aydi, Abdelwaheb, Zairi, Moncef., Ben Dhia, Hamed, (2013): Municipal solid waste management in Tunis City, Tunisia, in: Waste Management 33, 1682–1684.

Baldassarri, Delia (2015): Cooperative Networks: Altruism, Group Solidarity, Reciprocity, and Sanctioning in Ugandan Producer Organizations. *American Journal of Sociology* 121(2): 355-395.

Banfield, Edward C. (1958): The Moral Basis of a Backward Society. New York: The Free Press.

Banfield, Edward C. (1974): The unheavenly city revisited. Boston: Little Brown and Co.

Barnes, Samuel, Kaase, Max et al. (1979): Political Action: Mass Participation in Five Western Democracies, London: Sage.

Berelson, Bernard, Lazarsfeld, Paul F. and McPhee, William, N. (1954). Voting: A Study of Opinion Formation in a Presidential Campaign. Chicago: Chicago University Press.

Boh, Elvis (2016): Tunisia struggles to solve waste management problems, Africanews, published on April 15, 2016, online available:

https://www.africanews.com/2016/04/15/tunisia-struggles-to-solve-waste-management-problems/, [15.09.2019].

Bridge, Gary (2002): The Neighborhood and Social Networks, CNR Paper 4, Bristol: ESRC Center for Neighborhood Research.

Burn, Shawn M. (1991): Social Psychology and the Stimulation of Recycling Behaviors: The Block Leader Approach, Journal of Applied Social Psychology, 21, 611-629.

Cantoni, Davide/ Yang, David Y./ Yuchtman, Noam/ Zhang, Jane Y. (2019): Protests as Strategic Games: Experimental Evidence from Hong Kong's Antiauthoritarian Movement, The Quarterly Journal of Economics, 134(2), 1021-1077.

Carlsson, Frederik/ Johansson-Stenman, Olof/ Nam, Pham Khanh (2015): Funding a new bridge in rural Vietnam: a field experiment on social influence and default contributions, Oxford Economic Papers, 67(4), 987-1014.

Chennaoui, Henda (2019): Campagnes de Nettoyage, une Réappropriation de l'Espace public, Inkyfada, published on October 26, 2019, online available: https://inkyfada.com/fr/2019/10/26/tunisie-campagne-nettoyage/, [29.11.2019].

Cialdini, Robert B./ Goldstein, Noah J. (2004): Social Influence: Compliance and Conformity, Annu. Rev. Psychol. 55: 591-621.

Corstange, Daniel (2016): The Price of a Vote in the Middle East: Clientelism and Communal Politics in Lebanon and Yemen, Cambridge: Cambridge University Press.

Côté, Stéphane/ House, Julian/ Willer, Robb (2015): High economic inequality leads higher-income individuals to be less generous, PNAS, 112(52), 15838-15843.

Delhey, Jan, and Kenneth Newton (2003): Who trusts? The origins of social trust in seven societies, European Societies 5 (2): 93-137.

Díaz-Cayeros, Alberto/ Estevez, Federico/ Magaloni, Beatriz (2016): The Political Logic of Poverty Relief: Electoral Strategies and Social Policy in Mexico, Cambridge: Cambridge University Press.

Dietze, Pia/ Knowles, Eric D. (2016): Social Class and the Motivational Relevance of Other Human Beings. Evidence from Visual Attention, Psychological Science, 27(1), 1517-1527.

Dixit, Avinash/ Londregan, John (1996): The determinants of success of special interests in redistributive politics. the Journal of Politics, 58(4), 1132-1155.

Fahmy, Eldin, Williamson, Emma, & Pantazis, Christina (2016): Evidence and policy review: Domestic violence and poverty. Joseph Rowntree Foundation.
Filmer, Deon/ Pritchett, Lant H. (2001): Estimating wealth effects without expenditure data- or tears: an application to educational enrollments in states of India, Demography, 38, 115-132.

Foroudi, Layli (2019): As garbage piles up in Tunisian cities, waste pickers demand recognition, Reuters, published on August 1, 2019, online available: https://www.reuters.com/article/us-tunisia-waste-rights/as-garbage-piles-up-in-tunisian-cities-waste-pickers-demand-recognition-idUSKCN1UR300, [15.09.2019].

Gerber, Alan S./ Green, Donald P./ Larimer Christopher W. (2008): Social pressure and voter turnout: evidence from a large-scale field experiment, Am. Polit. Sci. Rev., 94(3), 653-63.

Giles, Michael W./ Dantico, Marilyn K. (1982): Political Participation and Neighborhood Social Context Revisited, American Journal of Political Science, 26(1), 144-150.

Gouldner, Alvin (1960): The Norm of Reciprocity: A Preliminary Statement. American Sociological Review 25, 161-178.

Guerrero, Lilliana Abarca/ Maas, Ger/ Hogland, William (2013): Solid waste management challenges for cities in developing countries, Waste Management 33, 220-232.

Hoffman, Steven M./ High-Pippert, Angela (2010): From private lives to collective action: Recruitment and participation incentives for a community energy program, Energy Policy 38, 7567-7574.

Huang, Chien-Chien/ Laing, Derek/ Wang, Ping (2004): Crime and Poverty: A Search-Theoretical Approach, International Economic Review, 45(3).

Huckfeldt, Robert (1979): Political Participation and the Neighborhood Social Context, American Journal of Political Science, 23(3), 579-592.

Huckfeldt, Robert/ Sprague, John (1987): Networks in Context: The Social Flow of Political Information, The American Political Science Review, 81(4), 1197-1216.

Kenny, Christopher B. (1992): Political Participation and Effects from the Social Environment, American Journal of Political Science, 36(1), 259-267.

Kersting, Norbert/ Sperberg, Jaime (2003): Political Participation, in: Berg-Schlosser, Dirk/ Kersting, Norbert (Eds.): Poverty and Democracy: Self-Help and Political Participation in Third World Cities, London and New York: Zed Books, 153-180.

Kraus, Michael W./ Rheinschmidet, Michelle L./ Piff, Paul K (2012): The intersection of resources and rank: signaling social class in face-to-face ecnounters. In Facing Social Class: How Societal Rank influences Interaction. Edyted by Fiske, Susan T./ Markus, Hazel Rose/ Russel Sage; 152-172.

Kraus, Michael W./ Piff, Paul K./ Keltner, Dacher (2009): Social class, sense of control and social explanation, J. Pers. Soc. Psychol., 97, 992-1004.

Lachman, Margie E./ Waever, Suzanne L. (1998): The sense of control as a moderator of social class differnces in health and well-being, J. Pers. Soc. Psychol. 1998, 74, 763-773.
Lake, Ronald La Due/ Huckfeldt, Robert (1998): Social Capital, Social Networks, and Political Participation, Political Psychology.

Lazarsfeld, Paul F., Berelson, Bernard and Gaudet, Hazel (1944): The People's Choice. New York: Columbia University Press.
Lim, Chaeyoon (2008): Social Networks and Political Participation: How Do Networks Matter? Social Forces, 87(2), 961-982.

McAdam, Doug/ Paulsen, Ronnelle (1993): Specifying the Relationship Between Social Ties and Activism, American Journal of Sociology, 99(3), 640-667.

McClurg, Scott (2003): Social Networks and Political Participation: The Role of Social Interaction in Explaining Political Participation, Political Research Quarterly, Vol. 56, 448-464.

Minghua, Zhu/ Xiumin, Fan/ Rovetta, Alberto/ Qichang, He/ Vicentini, Federico/ Bingkai, Liu/ Giusti, Alessandro/ Yi, Liu (2009): Municipal solid wate management in Pudong New Area, China, Waste Management 29, 1227-1233.

Operario, D./ Adler, N. E./ Williams, D. R. (2004). Subjective social status: Reliability and predictive utility for global health. Psychology & Health, 19, 237–246.

Nielsen, J. M., & Ellington, B. L. (1983). Social processes and resource conservation: A case study in low technology recycling. *Environmental psychology: Directions and perspectives*, 288-311.
Pavlova, Maria K./ Silbereisen, Rainer K. (2015): Supportive Social Contexts and Intentions for Civic and Political Participation: An Application of the Theory of Planned Behavior, Journal of Community & Applied Social Psychology, 25, 432-446.

Pichler, Florian, Wallace, Claire Denise (2009): Social Capital and Social Class in Europe: The Role of Soical Networks in Social Stratification, European Sociological Review 25(3), 319-332.

Piff, Paul K./ Robinson, Angela R. (2017): Social class and prosocial behavior: current evidence, caveats, and questions, Current Opinion in Psychology, 18, 6-10.

Piff Paul K./ Kraus, Michael W./ Côté, Stéphane/ Cheng, Bonnie H./ Keltner, Dacher (2010): Having less, giving more: the influence of social class on prosocial behavior. Journal of Personality and Social Psychology, 99, 771-784.

Pinkster, Fenne M. (2007): Localised Social Networks, Socialisation and Social Mobility in a Low-income Neighbourhood in the Netherlands, Urban Studies, 44(13), 2587-2603.

Putnam, Robert (2000): Bowling Alone: The Collapse and and Revival of American Community, New York: Simon & Schuster.

Putnam, Robert (1995): Turning In, Turning Out: The Strange Disappearance of Social Capital in America, Political Science and Politics, 28(4), 664-683.

Robinson, Angela R./ Piff, Paul K. (2017): Deprived, but not depraved: Prosocial behavior is an adaptive response to lower socioeconomic status, Behavioral and Brain Sciences, 40.

Rolfe, Meredith (2012): Voter Turnout: A Social Theory of Political Participation, Cambridge University Press, Cambridge.

Schaub, Max/ Gereke, Johanna/ Baldassarri, Delia (2020): Does Poverty Undermine Cooperation in Multiethnic Settings? Evidence from a Cooperative Investment Experiment. Journal of Experimental Political Science 7(1): 27–40.

Schultz, P. Wesley., Oskamp, Stuart/ Mainieri, Tina (1995): Who recycles and when? A review of personal and situational factors, Journal of Environmental Psychology 15, 105-121.

Scott, James C. (1976): The Moral Economy of the Peasant: Rebellion and Subsistence in Southeast Asia, New Haven and London: Yale University Press.

Siegel, David A. (2009): Social Networks and Collective Action. American Journal of Political Science, 53(1), 122–138.

Sinclair, Betsy/ McConnell, Margaret/ Michelson, Melissa R. (2013): Local Canvassing: The Efficacy of Grassroots Voter Mobilization. Political Communication, 30:1, 42-57.

Sinclair, Betsy (2012): The Social Citizen: Peer networks and political behavior. Chicago: University of Chicago Press.

Stephens, Nicole M./ Markus, Hazel Rose/ Townsend, Sarah S. M. (2007):

Choice as an Act of Meaning: The Case of Social Class, Journal of Personality and Social Psychology, 93(5), 814-830.

Tingsten, Herbert (1937). Political Behavior: studies in election statistics, London: P.S. King, Stockholm: Norstedt distr.

Tucker, Peter (1999): Normative Influences in Household Waste Recycling, Journal of Environmental Planning and Management 42(1), 63-82.

Tunisie numérique (2012): Tunisie: Les habitants de Makthar mènent une campagne de nettoyage, Tunisie numérique, published on January 19, 2012, online available: https://www.tunisienumerique.com/tunisie-les-habitants-de-makthar-menent-une-campagne-de-nettoyage/97545, [10.10.2018].

Uslaner, Eric M./ Brown, Mitchell (2005): Inequality, Trust, and Civic Engagement, American Politics Research, 33 (6), 868-894.

Van Eijk, Gwen (2010): Unequal networks: Spatial segregation, relationships and inequality in the city, Delft: Delft University Press.

Verba, Sidney/ Schlozman, Kay Lehman/ Brady, Henry E. (1995): Voice and Equality. Civic Voluntarism in American Politics. Cambridge/ London: Harvard University Press.

Verba, Sidney/ Nie, Norman H./ Kim, Jae-On (1978): Participation and Political Equality: A Seven-Nation Comparison, Chicago/ London: The University of Chicago Press.

Verba, Sidney/ Nie, Norman H. (1972): Participation in America: Political Democracy and Social Equality, New York, Evanston, San Francisco, London: Harper & Row, Publishers.

Vyas, Seema/ Kumaranayake, Lilani (2006): Constructing socio-economic status indices: how to use principal components analysis, Health Policy and Planning, 21(6), 459-468.
