# Supplementary material for: Neighborly social pressure and collective action: Evidence from a field experiment in Tunisia
Source: PLoS One. 2024 Jul 19;19(7):e0304269. doi: 10.1371/journal.pone.0304269 (PMC11259251; doi:10.1371/journal.pone.0304269)
Supplement: S4 File — (PDF) [file pone.0304269.s017.pdf]

Supplementary Material – S4

Consent Forms and Flyers

Participant Information (will be given to Heads of Household)

Research Project: Community Participation

Responsible Researcher:

The interviewer does not represent the government or any political party. He/She is working with a team of researchers from *anonymized*.

You are invited to participate in surveys and a field experiment. The field experiment includes clean-up event in your neighborhood in which you and your neighbors will be invited. The aim of this project is to understand when and why people participate in community activities.

We would like to start by asking you a few questions about your neighborhood and community activities in this neighborhood, especially, neighborhood clean-ups. We will also ask you a few personal questions, for example, about your employment status and your age. This will take approximately 10 minutes. Participation in the survey does not require you to join the community event.

Your answers are confidential and all information we collect in this project will be anonymized and stored on secured servers. <u>There won't be any negative consequences for you if you refuse to participate and you can decide to drop out at any time.</u> We would like your opinion with the knowledge that there are no right or wrong answers to the survey questions.

We will organize a clean-up event in your neighborhood on Sunday. During the clean-up event, we will provide trash bags, hand sanitizers and masks to all participants. The aim is to clean up the beach. We will also organize a lottery in which every participant has a chance to win small prizes. If you decide to come to the event, we will ask you a few additional questions about the reasons that you have participated and the neighbors you have invited to join. This won't take more than 5-10 minutes.

We would also like to ask you for your phone number so that we could contact you after the event if you decided not to show up. In that case, we would like to ask you a few additional questions over the phone to understand the reason for your decision to abstain. This won't take more than 5 minutes. If you join the event, we will not call you afterwards. All phone number will be deleted one week after the event. The phone numbers are not accessible to the researcher. They are collected solely to understand why some people may decide not to join the event.

If you have questions about the research project, you will find the contact information of the researchers and the interviewer firm in Tunis that is conducting this study below.

Participant Information (will be given to all additional participants of the clean-up event)

Research Project: Community Participation

Responsible Researcher:

The interviewer does not represent the government or any political party. He/She is working with a team of researchers from *anonymized*.

You are invited to participate in surveys and a field experiment. The field experiment includes this clean-up event in your neighborhood in which you and your neighbors were invited. The aim of this project is to understand when and why people participate in community activities.

We would like to start by asking you a few questions about your neighborhood and community activities in this neighborhood, especially, neighborhood clean-ups. We will also ask you a few personal questions, for example, about your employment status and your age, and your decision to join the event. This will take approximately 12 minutes.

Your answers are confidential and all information we collect in this project will be anonymized and stored on secured servers. <u>There won't be any negative consequences for you if you refuse to participate and you can decide to drop out at any time.</u> We would like your opinion with the knowledge that there are no right or wrong answers to the survey questions.

If you have questions about the research project, you will find the contact information of the researchers and the interviewer firm in Tunis that is conducting this study below.

Informed Consent Form (To Heads of Household)

Title of Research: Community Participation in Tunisia

Researcher:

Before agreeing to participate in this research study, it is important that you read the following explanation of this study. This statement describes the purpose, procedures, benefits, risks, discomforts, and precautions of the program. Also described are the alternative procedures available to you, as well as your right to withdraw from the study at any time. No guarantees or assurances can be made as to the results of the study.

Explanation of Procedures: This research is designed to investigate participation in community events such as neighborhood clean-ups. Participation in the study involves completion of a short survey and a follow up survey in one week. You will also be invited to join a community event. The surveys will be conducted by the interviewer through a tablet computer.

Risks and Discomforts: Potential risks or discomforts include a range of emotional feelings when asked questions during the interview about your socioeconomic status, employment history, and membership in organizations. You may also feel discomfort and risk of infection when participating with others in a community event. We will provide masks, gloves, hand sanitizers and trash bags for all participants to minimize the risk of infection. We also ask you to collect trash in groups of only three people and to please keep distance to other participants.

Benefits: The anticipated benefit of participation is the opportunity to contribute to the community by joining the community event and to discuss any issues that arise in our everyday life.

Confidentiality: The information gathered during this study will remain confidential. Only the researcher and the enumerator firm will have access to the study data and information. The data will be anonymized and stored on secured servers. The results of the research will be published in the form of a paper and may be published in a professional journal or presented at professional meetings. It will not be possible to identify individual participants from the anonymized data and the analysis presented in the research output."

Withdrawal: Participation in this study is voluntary; refusal to participate will not involve any negative consequences. Each participant is free to withdraw consent and discontinue participation in this project at any time. Participants can also decide not to respond to those questions of the survey that they feel uncomfortable answering.

Payment: You will not be compensated in any way for participation in this study. Yet, we will organize a lottery during the community event in which you can win small prizes.

Questions: You will receive additional information on this project in print, including the contact details of the researcher and enumerator firm. Any questions concerning the research project, including questions regarding rights as a person in this research project, should be directed to (anonymized).

Agreement

This agreement states that you have received a copy of this informed consent. Your signature below indicates that you agree to participate in this study.

Signature of Subject: ___________________________ Date:_______

Subject name (printed):_________________________

Informed Consent Form (To All Additional Participants of the Clean-up Event)

Title of Research: Community Participation in Tunisia

Researcher:

Before agreeing to participate in this research study, it is important that you read the following explanation of this study. This statement describes the purpose, procedures, benefits, risks, discomforts, and precautions of the program. Also described are the alternative procedures available to you, as well as your right to withdraw from the study at any time. No guarantees or assurances can be made as to the results of the study.

Explanation of Procedures: This research is designed to investigate participation in community events such as neighborhood clean-ups. Participation in the study involves completion of a short survey and a follow up survey in one week. You will also be invited to join a community event. The surveys will be conducted by the interviewer through a tablet computer.

Risks and Discomforts: Potential risks or discomforts include a range of emotional feelings when asked questions during the interview about your socioeconomic status, employment history, and membership in organizations. You may also feel discomfort and risk of infection when participating with others in a community event. We will provide masks, gloves, hand sanitizers and trash bags for all participants to minimize the risk of infection. We also ask you to collect trash in groups of only three people and to please keep distance to other participants.

Benefits: The anticipated benefit of participation is the opportunity to contribute to the community by joining the community event and to discuss any issues that arise in our everyday life.

Confidentiality: The information gathered during this study will remain confidential. Only the researcher and the enumerator firm will have access to the study data and information. The data will be anonymized and stored on secured servers. The results of the research will be published in the form of a paper and may be published in a professional journal or presented at professional meetings. It will not be possible to identify individual participants from the anonymized data and the analysis presented in the research output."

Withdrawal: Participation in this study is voluntary; refusal to participate will not involve any negative consequences. Each participant is free to withdraw consent and discontinue participation in this project at any time. Participants can also decide not to respond to those questions of the survey that they feel uncomfortable answering.

Payment: You will not be compensated in any way for participation in this study.

Questions: You will receive additional information on this project in print, including the contact details of the researcher and enumerator firm. Any questions concerning the research project, including questions regarding rights as a person in this research project, should be directed to (anonymized).

Agreement

This agreement states that you have received a copy of this informed consent. Your signature below indicates that you agree to participate in this study.

Signature of Subject: ___________________________ Date:_______

Subject name (printed):_________________________

Informed Consent Form (Collection of Phone Numbers, To Heads of Household only)

Title of Research: Community Participation in Tunisia

Researcher:

Thank you for participating in our survey.

We would like to ask you for your phone number so that we could contact you after the event if you decided not to participate in our clean-up event. If you decide to abstain, we would like to ask you a few additional questions over the phone to understand the reason for your decision. This won't take more than 2 minutes. If you join the event, we will not call you afterwards. All phone number will be deleted one week after the event. The phone numbers are not accessible to the researcher. They are collected solely to understand why some people may decide not to join the event and you can decide to withdraw at any time.

If you agree that we can contact you after the event, please write down your name and phone number: *(Enumerator will be allowed to help with writing down the printed name of the respondent and the phone number)*

Subject name (printed): ________________________

Phone Number: ________________________

Questions: You will receive additional information on this project in print, including the contact details of the researcher and enumerator firm. Any questions concerning the research project, including questions regarding rights as a person in this research project, should be directed to (anonymized).

Figure 1. Flyer for Treatment (neigbor recruiter) and Placebo Group (outsider recruiter)

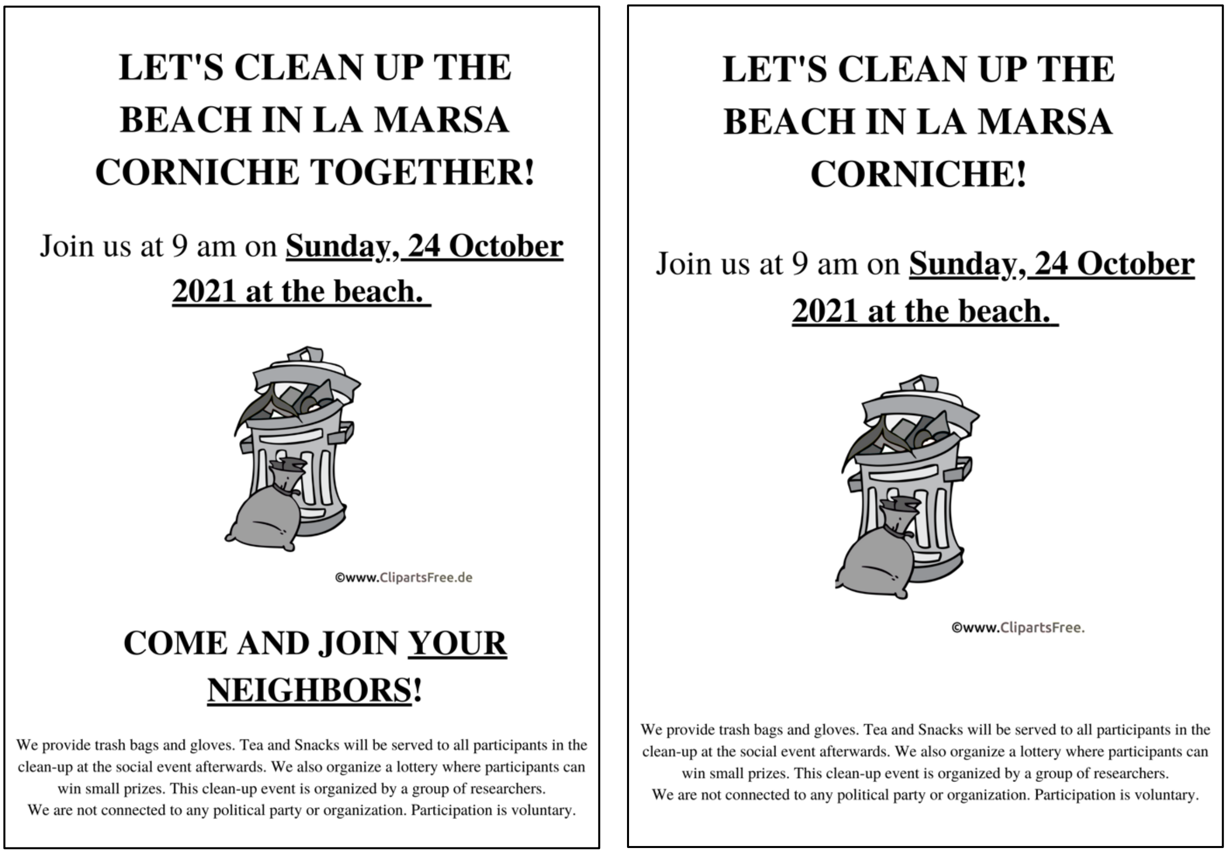

Note: The flyers were translated in Arabic. We updated the dates in the Arabic versions of the flyers. Cleanups were held on 14 and 21 November 2021.

Script for Enumerators after Conducting the Survey

”The second enumerator joins after the survey is conducted (local or non-local recruiter)

“(The second enumerator joins the meeting with flyers and a button) My name is {first name of enumerator} and I am {first name of enumerator} colleague. I live in {insert name of the neighborhood/another area of Tunisia}, {we are neighbors/ I am not from this neighborhood}. We would like to organize a clean-up event in this neighborhood next Sunday and we would like your help. You have been randomly selected to become a “community mobilizer” for this clean-up event. All information we collect will be anonymized. There is no penalty for refusing to participate in the event and you can decide to leave the event at any time. If you come to the event, we would like to ask you a few questions after the cleanup which won’t take more than 10 minutes.

During the event, we will provide trash bags, hand sanitizers and masks to all participants. The aim is to clean up the beach in this neighborhood. We also organize a lottery in which every participant has a chance to win small prizes.

If you are interested, {we/me, as your neighbor,}would be happy to see you next week!”
