# Supplementary material for: Neighborly social pressure and collective action: Evidence from a field experiment in Tunisia
Source: PLoS One. 2024 Jul 19;19(7):e0304269. doi: 10.1371/journal.pone.0304269 (PMC11259251; doi:10.1371/journal.pone.0304269)
Supplement: S5 File — (PDF) [file pone.0304269.s018.pdf]

Supplementary Material – S5

Neighborhood Selection and Sampling Method

Social Neighborhood Context and the Selection of the Neighborhoods

In a first round of preliminary fieldwork in November 2019, I conducted semi-structured interviews with citizens, civil society activists and politicians in different neighborhoods in Tunis (please see the appendix for adetailed list of neighborhoods considered in table C2). Different municipalities within the governorate of Tunis were preselected by comparing municipality level data from the INS census 2014. I compared municipalities based on unemployment rates, analphabetism and other indicators (see table C1).

I also relied on the local knowledge of our research assistant who is based in Tunis, our contact person from a local survey firm in Tunis, and other informants from different local civil society organizations and a political foundation working, more specifically, on environmental protection in Tunisia to preselect the municipalities and neighborhoods within each municipality.

After this fieldwork, I narrowed down our choice of neighborhoods (see table C1-2 for reasonings for this selection). The neighborhoods that I may want to include in the actual experimental study are Le Kram - West as a homogeneously poor neighborhood, La Marsa Plage as wealthy neighborhood and Casino in La Goulette as a mixed neighborhood. I received data including neighborhood development indicators from INS to assess the socioeconomic composition of the neighborhoods that are listed as well as other factors we will use to ensure comparability between these neighborhoods. The data is based on data from the 2014 Census as well as additional data from consumptions and employment surveys that were conducted by the INS in recent years.

Table 1. Municipality level data (INS 2014)

Governorate	Municipality	Population numbers	Age (mean)	Highest level of education (population >/=10 years) in percent	No formal education (population >/=10 years) in percent	Analphabetism (population >=10 years) in percent	Unemploy ment (ratio)	Unemployment among people with high school education (ratio)	Unemployment among University graduates (ratio)	Housing (5 rooms or more) in percent	Housing (villa or duplex) in percent
Tunis	Sidi Hassine	109672	30.3	7.0	9	15.6	16.5	10.6	27.23	3.3	7
Tunis	Le Kram	74132	33.1	25.4	3.9	9	11.6	41.6	8.9	7.6	16.2
Tunis	La Goulette	45711	32.7	37.6	1.7	5.2	8.7	57.6	6.8	7.1	21.3
Tunis	Medina	21400	37	12.2	9.3	13.7	14.03	18.4	17.5	8.3	2.4
Tunis	La Marsa	92987	34.2	25.2	6.1	9.3	9	37	8.2	12.6	36.6
Ariana	La Soukra	129693	30.9	22	10.5	10.5	10.4	34.1	10.2	9.6	24.2
Tunis	Carthage	24216	36	26.5	3.6	8.7	10.9	40.8	10.1	16.1	33.2
--	TUNIS (total)	1056247	-	19.8	4.9	-	18.6	32.5	12.5	8.2	15.1
--	TUNISIA (total)	7437551	31.4	15.5	6.1	19.3	21.4	25.5	18.3	8.7	25.9

Source: National Census 2014, National Statistics Institute (INS 2016).

Table 2. List of Neighborhoods Considered and Socioeconomic Composition

Governorate	Municipality	Neighborhood	Socioeconomic Composition
Tunis	Sidi Hassine	Centre ville de Sidi Hassine	Lower Middle Class
Tunis	Sidi Hassine	25 Juillet	Lower Class
Tunis	Sidi Hassine	20 Mars	Middle Class
Tunis	Le Kram	5 Décembre	Lower Class and Lower Middle Class
Tunis	La Goulette	Casino	Mixed
Tunis	La Goulette	Kheireddine	Mixed
Tunis	Medina	Medina (Souk pour les femmes)	Mixed
Tunis	La Marsa	Corniche	Upper Class
Ariana	La Soukra	Chotrana III	Upper Class
Tunis	Carthage	Dermech	Upper Class

Figure 1. Pictures of Selected Neighborhoods and Clean-

Le Kram West (Poor Neighborhood)

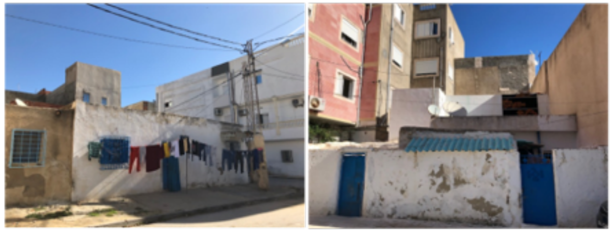

La Goulette Casino (Mixed Neighborhood)

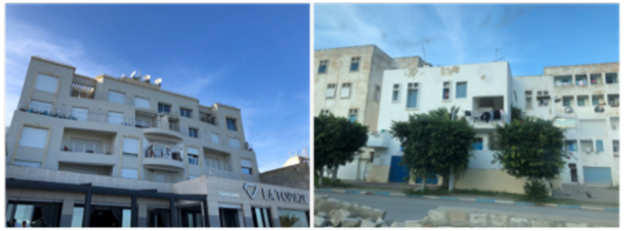

La Marsa – Corniche (Wealthy Neighborhood)

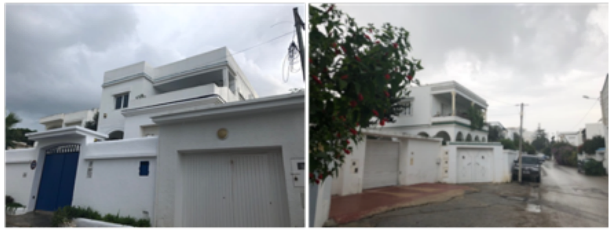

Clean-up Site: Beach

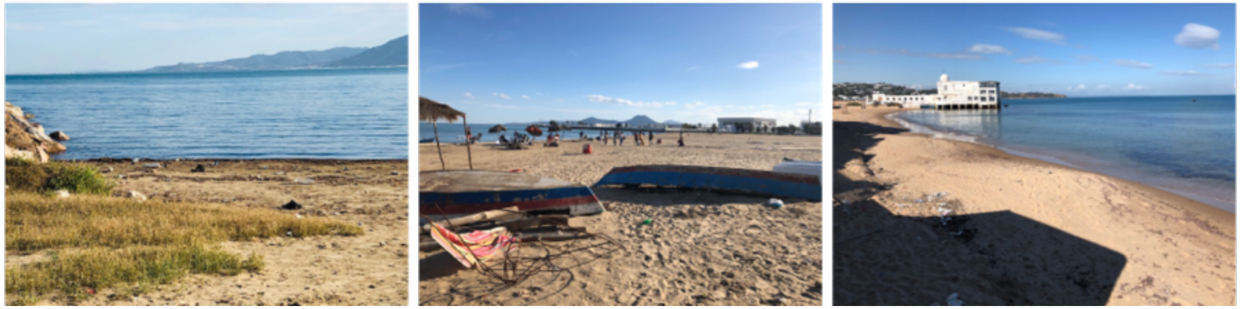

Note: Le Kram West, La Goulette Casino and la Marsa Corniche are neighborhoods of Tunis. The neighborhoods are located along the coastal line and are located next to each other.

Figure 2. Picture of Neighborhood Clean-up Event in La Marsa

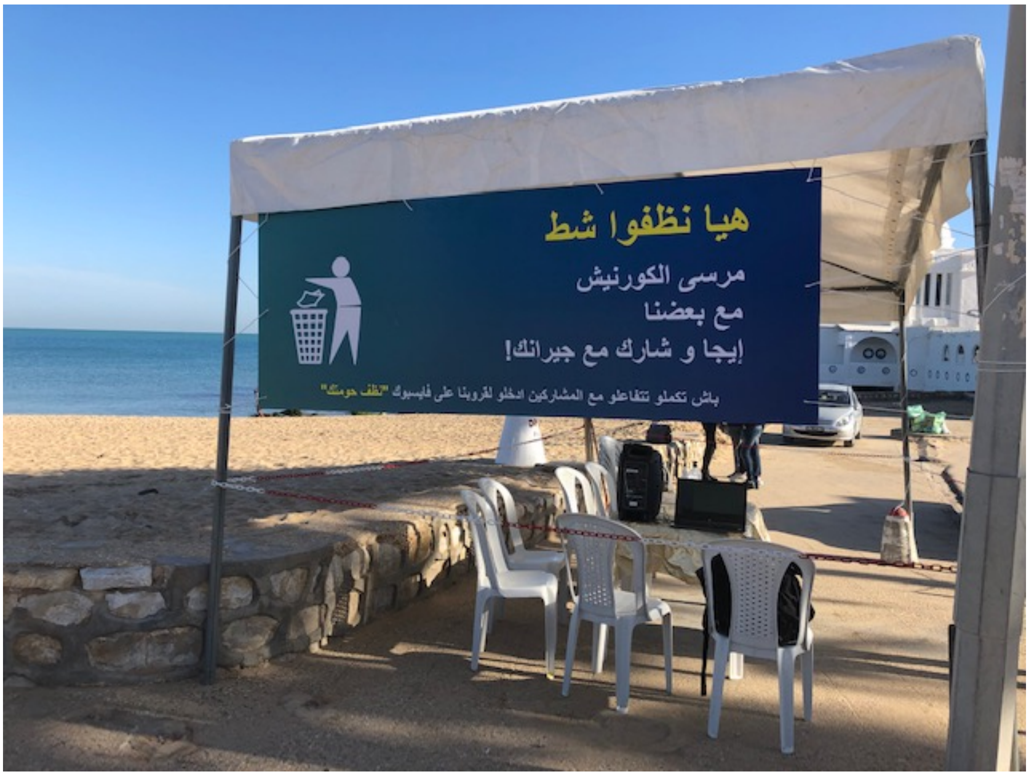

Figure 3. Picture of Cleanup Event in La Goulette

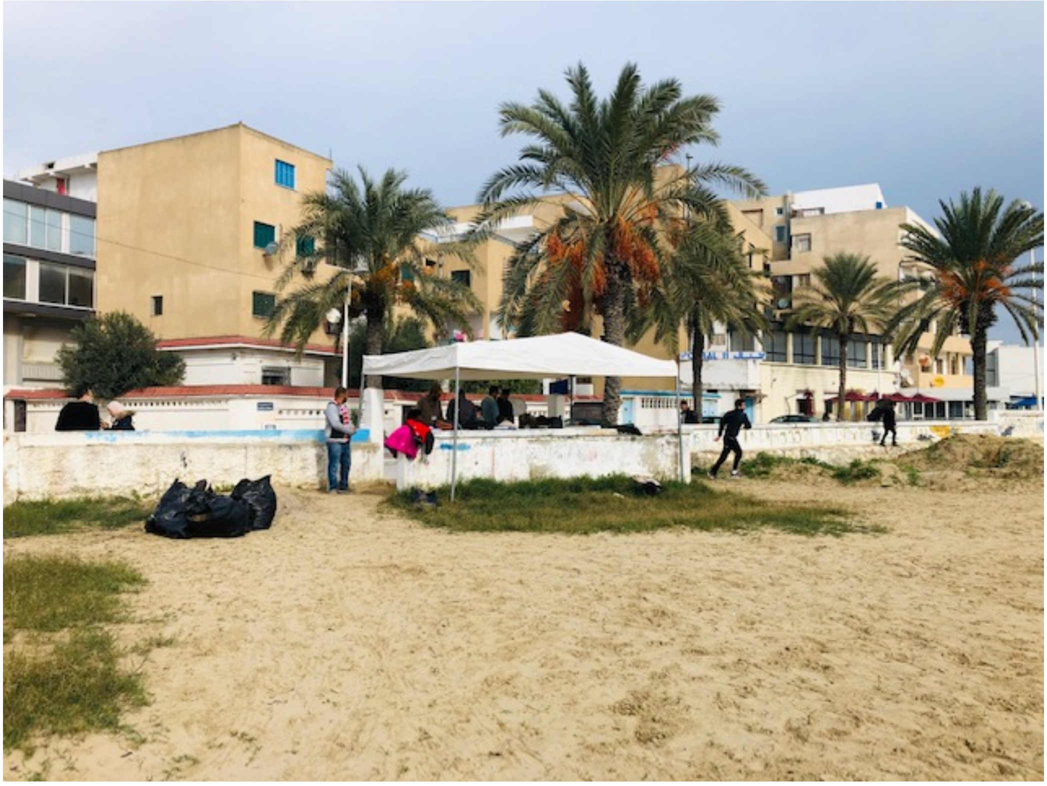

Figure 4. Picture of Cleanup Event in Le Kram

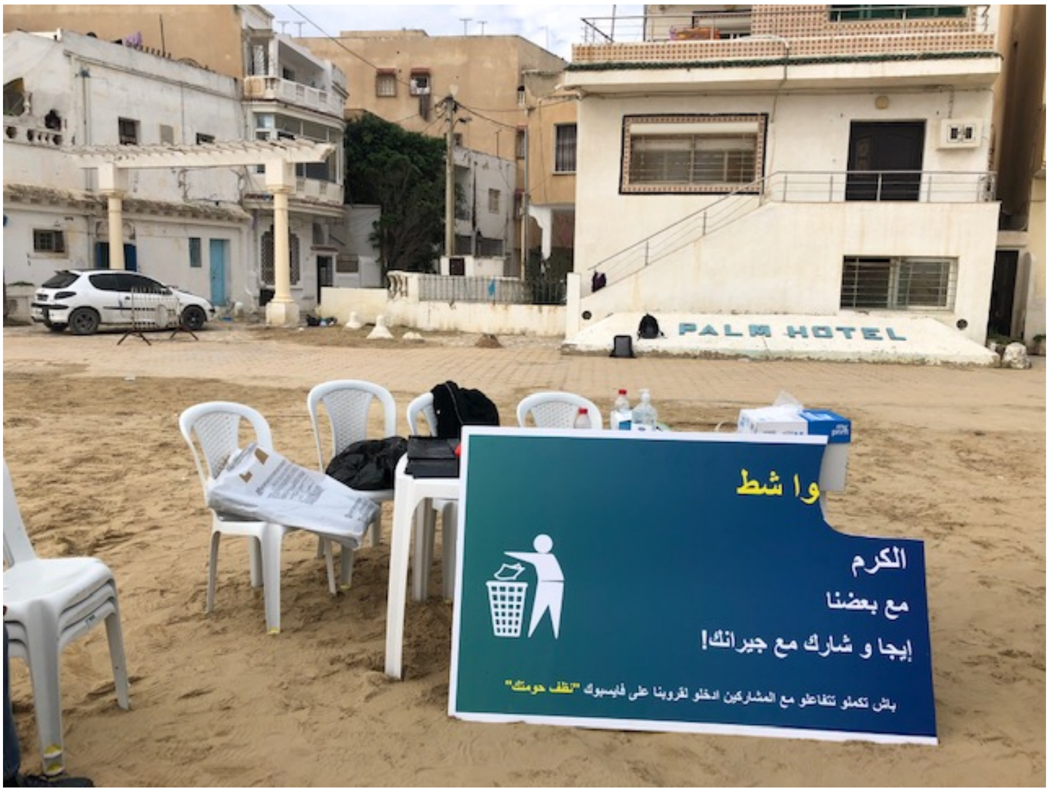

Note: Sign was broken during transportation.

Figure 5. Filled Trash Bags in La Goulette

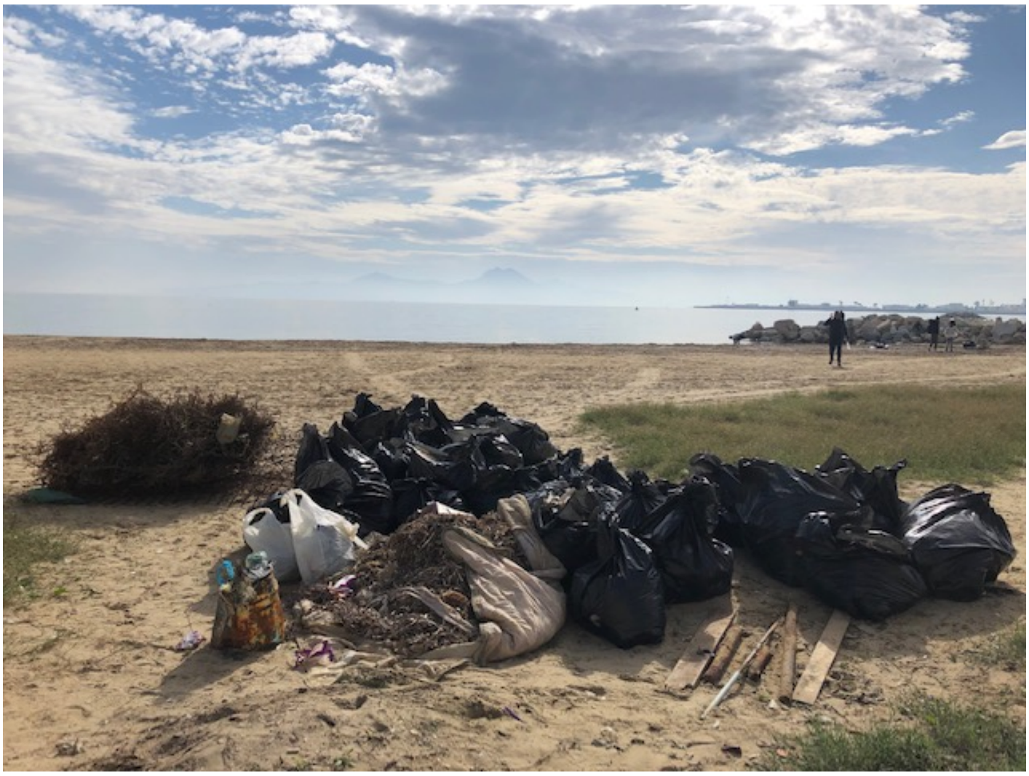

Table 3. Summary of Interviews and Neighborhood Selection during Pre-liminary Fieldwork in November 2019

District	Neighborhood	Day	Category	Impression	Interview partners	Clean-up side	Concerns
Sidi Hassine	20 mars	04-Nov	middle class	middle class, maybe also lower middle class but relatively speaking one of the better neighborhoods in Sidi Hassine; still a lot of trash along the streets and on private property where there are no houses at the moment	group of students (high school and mid-20s university students) and activists from this neighborhood who organized clean-up events in their neighborhood/ painted walls etc.	health center (area around the building)	relatively wealthy (middle class) neighborhood and the area around the health center is usually closed, so that there is not as much trash as outside on the street
Sidi Hassine	Centre ville de Sidi Hassine	04-Nov	middle class/ lower middle class	a lot of shops and markets at day time (very busy); houses are middle class not necessarily extremely poor in the city center	Sidi Hassine page activists who organized clean-up events in the city center and the cemetery where they collected equipement and money from the citizens first and then met for a clean-up	Cemetery	was cleaned up end of October so that it was clean when we vistited but would be a great area to clean up in Sidi hassine
Le Kram	El Stih (neighborhood in 5 Décembre 4 in total within this one)	05-Nov	poor/lower middle-class	poor neighborhood, people keep their streets and the area before their houses very clean in this neighborhood (Brooms everywhere)	4 interviews with mostly elderly people between 40-60+)	Aéroport park close to the neighborhood	not direclty at the neighborhood (was not extremely dirty but one part of it was but need to see if it can be cleaned; stones etc.)
La Goulette	Casino	05-Nov	mixed (very wealthy and lower class)	very mixed with restaurants by the sea but also very poor people who live from 200 Dinar per month	3 interview partner (one lives in Khereddine but works in Casino and has little grocery shops there)	Beach	lot of tourists around in summer but should not be a problem in Spring
La Goulette	Kheireddine	05-Nov	mixed (very wealthy and lower class)	similar to Casino	one person who lives here but works in Casino	Beach	interviewed people in casino, not here
Sidi Hassine	25 Juillet	06-Nov	poor	very poor, social housing (25 years ago built by the government after former housing that was build by the French was destroyed)	4 interview partners (one very poor women, one female shop owner , one old man and a 25-year old man)	foodball court/ area in the neighorhood	people liked to present themselves as rather isolated from their neighbors (older people) even though they seem to spend a lot of time on the streets even though it was raining; high crime rates
La Marsa	Corniche	06-Nov	wealthy	very wealthy, upper class Tunisians and some experts, but still trash at the beach and in some streets to the beach	4 interview partners (2 man in their 50s, a medical doctor (female) also in her 50s and a French woman in her late 30s)	Beach	may have a regular clean-up (more than other places) but still looked pretty polluted
Medina	Souk des femmes	07-Nov	mixed	very mixed even though 80 percent middle class but very rich shop owners and very poor people collecting plastic	5 interview partners (one shop owner, one artisan working with copper mugs; one berbecha who collects plastic bottles; one very rich diamond trader)	very clean in most parts because of cleaning campaigns; one parking spot/ place was very dirty	not enought places for clean-up if the events continue
La Soukra	Chotrana III	07-Nov	wealthy	very new houses, wealthy	1 medical doctor (just finished her studies)	a lot of places in the neighborhood are dirty	very new neighborhood, people moved there a few years ago and many houses are still under construction
Carthage	Dermech	07-Nov	wealthy/ very wealthy	very clean neighborhood with very expensive villas	3 interview partner (one rents a villa, one is a English teacher at the American high school and a third one was the son of one of the oldest families in Carthage; unemployed economist)	place at the beach, Carthage Punic port but was recently cleaned)	a lot of diplomats (foreigners) live in the neighborhood

Note: All interviews were conducted in Arabic and French by the author and a research assistant. Most interviews were recorded. In few instances, we did not receive permission from the respondents to record the interview. We took handwritten notes during these interviews instead.

Sampling Method

After randomly selecting the sampling points, we draw the PSUs (virtual drawing). The PSUs are a group of households (one or more blocks) closest to the selected sampling points. The determination of the block where we conducted the survey was done in the field using the block most close to the starting point. The starting point can be a main street or a shop.

We randomly selected the household using the random step method (selecting one household and skipping the next 2 houses). Starting point in each point/cell: 1st house on the right, next target is 4th house on the right, so the interviewer go through the whole block and get back to the starting point.

In some areas, the step method has not always been respected, and this was due to the dispersion of houses and the limited number of houses. So, enumerators and their team leaders did the horizontal random selection or another random way to select houses. The enumerators went in different directions from the starting point. The number of ways depended on the location of the starting point. If it is surrounded by 4 residential communities (concentrations) (right, left, the north and the south of the starting point), each interviewer takes the way leading to one of the four communities. If the team didn't reach the targeted number of households, it reconducted the random step with the remaining houses in the sampling point. One household is contacted by two enumerators (one is a neighbor and the other is a strange or two are strangers).
