# Supplementary material for: Neighborly social pressure and collective action: Evidence from a field experiment in Tunisia
Source: PLoS One. 2024 Jul 19;19(7):e0304269. doi: 10.1371/journal.pone.0304269 (PMC11259251; doi:10.1371/journal.pone.0304269)
Supplement: S6 File — (PDF) [file pone.0304269.s019.pdf]

Supplementary Material – S6

Hypotheses Testing

Table 1. Full List of Hypotheses

**Hypothesis**
H1a: On average, neighbor recruiters will successfully recruit more participants to the clean-up events than community outsiders. I measure this by presence at the clean-up event first among the sample of initial HoH recruiters, and second, among their subsequent recruits.
H1b: Respondents will be more likely to participate when they have been recruited by a neighbor than by a community outsider. I measure this by presence at the clean-up event first among the sample of initial HoH recruiters, and second, among their subsequent recruits. I will measure this by presence at the clean-up event first among the sample of initial HoH recruiters, and second, among their subsequent recruits.
H1c: Respondents will collect more trash/ be more likely to wear the button during the clean-ups when they have been recruited by a neighbor than by a community outsider.
H2a: In a post-treatment survey, we expect those who were recruited by neighbors to have higher individual scores on community mechanisms than those recruited by community outsiders (those not at the event will be called and asked to complete a follow-up survey on why they did not participate). This effect is expected to hold across both the sample of initial HoH recruiters and their subsequent recruits.
H2b: I expect high scores on community mechanisms to increase the effect of neighbor recruitment on participation and the number of trash bags filled. I will use causal mediation analyses as described by Imai and Yamamoto (2013) to test if the proposed mechanisms drive the relationship between neighborly recruitment and participation.

H3a: Poorer individuals will be more likely to fear community sanctions and to be monitored by their neighbors, to have stronger feelings of obligation, and a stronger sense of duty to reciprocate with their neighbors than wealthier individuals with theirs. Wealthier individuals will have more generalized social trust than the poor but the poor will be more trusting in their neighbors than the wealthy. Note that these are observational analyses.
H3b: Assuming H3a is true, on average, poor participants will both be more likely to participate themselves and will be more likely to successfully recruit their neighbors for participation in the clean-up event and fill more trash bags/ wear the buttons compared to wealthier individuals when asked by a co-local rather than community outsiders to participate in the clean-ups.
H4a: Community mechanisms, with the exception of generalized trust, are stronger among members within the homogeneously poor neighborhood than the socioeconomically mixed neighborhood. (Observational analysis).
H4b: Participation in community initiatives as measured by participation among initial recruits (community mobilizers) and the participation rate/ the number of people who signed up for the facebook group will be higher among individuals living in homogeneously poor neighborhoods than those living in socioeconomically mixed ones. (Observational analysis).
H4c: Recruitment by neighbor mobilizers will drive higher participation in community initiatives and a stronger engagement (i.e. the number of trash bags filled, wearing a button or not) in homogeneously poor neighborhoods than in socioeconomically mixed (examined among initial recruits and by looking at the number of additional recruits by community mobilizer).
H5a: Poorer individuals specifically are more likely to participate in civic action when living in communities in which they constitute the socioeconomic majority than poorer individuals living in socioeconomically mixed neighborhoods (among initial recruits). (Observational analyses).
H5b: Poorer individuals specifically will be more successful in recruiting their neighbors when living in neighborhoods in which they constitute the socioeconomic majority than poorer individuals living in socioeconomically mixed neighborhoods (number of additional recruits by community mobilizer). (Observational analyses).

Note: Hypotheses were pre-registered.

Statistical Tests for Hypotheses 1c-4a and 5a-b

I have reported the findings for the main hypotheses 1a-b and 4a-b in the main text and appendix A. Below, I report the findings for the hypotheses 1c-4a and 5a-b (see list of pre-registered hypotheses in Appendix F) regarding community mechanisms and the moderating factor of individual poverty. Hypothesis 5b could not be tested empirically because respondents did not invite additional neighbors to the cleanups.

Table 1. Average Treatment Effects with Number of Trash Bags filled without controls (Hypothesis H1c)

	Model (1) Number of Trash Bags filled
Treatment	-0.488
	(1.009)
Constant	3.583**
	(0.802)
Observations	33
R2	0.0074

Note: *p<0.1 **p<0.05 ***p<0.01. Based on OLS regression. Standard errors in parentheses. Clustered standard errors on the neighborhood level in Model (1).

I do not find evidence that respondents who received the treatment condition filled more trash bags than their counterparts who received the placebo condition. However, very small numbers of observations (respondents who joined the cleanups) do not allow us to draw valid conclusion due to low statistical power.

Table 2. Obligation to Help your Neighbors by Treatment and Control Group and Individual Poverty (Hypotheses 2a, 3a and 4a)

	Treatment	Control	Poor Respondent	Wealthy Respondent	Poor Neighborhood	Mixed Neighborhood	Wealthy Neighborhood
Not much	6	1	4	2	2	3	2
	(28.57)	(7.69)	(21.05)	(22.22)	(40)	(15.79)	(20)
Somewhat	7	7	5	5	2	8	4
	(33.33)	(53.85)	(26.32)	(55.56)	(40)	(42.11)	(40)
Very much	7	4	9	1	1	6	4
	(33.33)	(36.36)	(47.37)	(11.11)	(20)	(31.58)	(40)
Do not know/ Refuse to answer	1	1	1	1	0	2	0
	(4.76)	(7.69)	(5.26)	(11.11)	(0)	(10.53)	(0)
Total	21	13	19	9	5	19	10
	(100)	(100)	(100)	(100)	(100)	(100)	(100)

Note: Absolut numbers reported. Percentages in parentheses. Responses to the following survey question are presented: "We would like to understand how people interact in your area. How much do you think people are obligated to help others, even if it costs them a day's wages? Would you say that the person is <1> not obligated at all to help, <2> somewhat obligated, or <3> very obligated, …<98> Don't know/Refuse to answer…if the person is a neighbor."

I do not find evidence that obligations to help neighbors would be higher in the treatment group than the placebo group, nor among poorer than wealthier respondents and neighborhoods. However, low participation rates in the cleanups do not allow us to draw valid statistical conclusions.

Table 3. Trust in Neighbors by Treatment and Control Group and Individual Poverty (Hypotheses 2a, 3a and 4a)

	**Treatment**	**Control**	**Poor Respondent**	**Wealthy Respondent**	**Poor Neighborhood**	**Mixed Neighborhood**	**Wealthy Neighborhood**
Distrust very much	1	1	1	1	0	0	2
	(5.88)	(7.69)	(5.26)	(11.11)	(0)	(0)	(20)
Distrust Somewhat	6	0	3	2	1	5	0
	(28.57)	(0)	(15.79)	(22.22)	(20)	(26.32)	(0)
Trust Somewaht	5	7	8	4	1	6	5
	(23.81)	(53.85)	(42.11)	(44.44)	(20)	(31.58)	(50)
Trust very much	8	5	7	2	3	7	3
	(38.10)	(38.46)	(36.84)	(22.22)	(60)	(36.84)	(30)
Do not know/ Refuse to answer	1	0	0	0	0	1	0
	(4.76)	(0)	(0)	(0)	(0)	(5.26)	(0)
Total	21	13	19	9	5	19	10
	(100)	(100)	(100)	(100)	(100)	(100)	(100)

Note: Absolut numbers reported. Percentages in parentheses. Responses to the following survey question are presented: “Please tell me how much you trust your neighbors. <1> distrust very much, <2> distrust somewhat, <3> trust somewhat , <3> trust very much and <98> Don’t know/Refuse to answer.”

I do not find evidence that obligations to help neighbors would be higher in the treatment condition than the placebo group, nor among poorer than wealthier respondents and neighborhoods. Individuals are more trusting in the placebo than treatment group, and among poorer than wealthier respondents. However, low participation rates in the cleanups do not allow us to draw valid statistical conclusions.

Table 4. Monitoring by Neighbors by Treatment and Control Group and Individual Poverty (Hypotheses 2a, 3a and 4a)

	**Treatment**	**Control**	**Poor Respondent**	**Wealthy Respondent**	**Poor Neighborhood**	**Mixed Neighborhood**	**Wealthy Neighborhood**
Yes	15	10	16	6	3	16	6
	(71.43)	(76.92)	(84.21)	(66.67)	(60)	(84.21)	(60)
No	5	2	2	2	2	3	2
	(23.81)	(15.38)	(10.53)	(22.22)	(40)	(15.79)	(20)
Do not know/ Refuse to answer	1	1	1	1	0	0	2
	(4.76)	(7.69)	(5.26)	(11.11)	(0)	(0)	(20)
Total	21	13	19	9	5	19	10
	(100)	(100)	(100)	(100)	(100)	(100)	(100)

Note: Absolut numbers reported. Percentages in parentheses. Responses to the following survey question are presented: “If an event is held in your neighborhood, do you think your neighbors keep track of whether you attend it? <1> yes, <2> no, <98> Don’t know/Refuse to answer.”

Monitoring appears to be more likely in the mixed neighborhood than the poor and wealthy neighborhoods and among poorer respondents than wealthier respondents in our sample. However, low participation rates in the cleanups do not allow us to draw valid statistical conclusions.

Table 5. Sanctioning by Neighbors by Treatment and Control Group and Individual Poverty (Hypotheses 2a, 3a and 4a)

	Treatment	Control	Poor Respondent	Wealthy Respondent	Poor Neighborhood	Mixed Neighborhood	Wealthy Neighborhood
Mean	5.82	6	6	5.67	6.5	5.85	5.5
(Std. Error)	(0.63)	(1.095)	(1.53)	(1.63)	(4.95)	(1.405)	(0.707)
Min	3	5	4	3	3	4	5
Max	10	8	8	8	10	8	6
Total	11	6	7	6	2	13	2

Note: Responses to the following survey question are presented: "On a scale from 0 to 10 where 0 means not at all and 10 very much, how much do you agree with the following statements: Your neighbors will punish you in some way (e.g., talk badly about you or your household) if you don't participate in community events."

Low participation rates in the cleanups and high non-response rates for the survey question do not allow us to draw valid statistical conclusions.

Table 6. Belonging by Treatment and Control Group and Individual Poverty (Hypotheses 2a, 3a and 4a)

	Treatment	Control	Poor Respondent	Wealthy Respondent	Poor Neighborhood	Mixed Neighborhood	Wealthy Neighborhood
Very close	3<br>(14.29)	4<br>(30.77)	4<br>(21.05)	2<br>(22.22)	2<br>(40)	4<br>(21.05)	1<br>(10)
Close	9<br>(42.86)	2<br>(15.38)	6<br>(31.58)	1<br>(11.11)	2<br>(40)	7<br>(36.84)	2<br>(20)
Not very close	5<br>(23.81)	6<br>(46.15)	4<br>(21.05)	6<br>(66.67)	1<br>(20)	4<br>(21.05)	6<br>(60)
Not close at all	4<br>(19.05)	1<br>(7.69)	5<br>(26.32)	0<br>(0)	0<br>(0)	4<br>(21.05)	1<br>(10)
Do not know/ Refuse to answer	0<br>(0)	0<br>(0)	0<br>(0)	0<br>(0)	0<br>(0)	0<br>(0)	0<br>(0)
Total	21<br>(100)	13<br>(100)	19<br>(100)	9<br>(100)	5<br>(100)	19<br>(100)	10<br>(100)

Note: Absolut numbers reported. Percentages in parentheses. Responses to the following survey question are presented: "How would you describe your feelings towards your neighbors? <1> very close, <2> close, <3> not very close , <4> not close at all, and <98> Don't know/Refuse to answer."

Relationships with neighbors appear to be stronger in the treatment than the control group. However, low participation rates in the cleanups do not allow us to draw valid statistical conclusions.

Table 7. Treatment Effects on Community Mechanisms (Hypothesis 2b)

	Model (1) Obligation to Help	Model (1) Trust	Model (1) Monitoring	Model (1) Sanctioning	Model (1) Belonging
Treatment	-0.2	4.293	-0.083	-0.182	0.168
	(0.228)	(2.389)	(0.209)	(0.452)	(0.328)
Constant	2.25***	3.231***	0.833**	6***	2.308**
	(0.073)	(0.199)	(0.153)	(0.298)	0.307
Observations	32	34	32	17	34
R2	0.0171	0.0169	0.0095	0.0026	0.0071

Note: *p<0.1 **p<0.05 ***p<0.01. Based on OLS regression. Standard errors in parentheses. Clustered standard errors by neighborhood.

Very small number of observations (respondents who joined the cleanups) does not allow to draw valid conclusion due to low statistical power.

Table 8. Heterogeneous Treatment Effects by Individual Wealth (Hypothesis 3b)

	Model (1) Participation
Treatment	-0.010
	(0.005)
Poor	0.005
	(0.008)
Treatment* Poor	0.017
	(0.023)
Constant	0.0226*
	(0.007)
Observations	1,103
R2	0.0025

Note: *p<0.1 **p<0.05 ***p<0.01. Based on OLS regression. Standard errors in parentheses. Clustered standard errors by neighborhood.

I do not find evidence in support of H3b.

Table 9. Treatment Effects by Neighborhood Context (Poor Sample only) (Hypothesis 5a)

	Model (1)
	Participation
Treatment	-0.015
	(0.021)
**Neighborhood (*Poor Neighborhood as Baseline*)**	
Mixed Neighborhood	0.004
	(0.026)
Wealthy Neighborhood	-0.011
	(0.022)
**Interaction**	
Mixed*Treatment	0.056*
	(0.033)
Wealthy*Treatment	0.052
	(0.038)
Constant	0.027
	(0.019)
Observations	698
R2	0.0169

Note: *p<0.1 **p<0.05 ***p<0.01. Based on OLS regression. Standard errors in parentheses. Clustered standard errors by neighborhood.

Poorer respondents who received the treatment condition appear to be more inclined to join the cleanups when living in the socioeconomically mixed neighborhood as compared to the poor neighborhood. Thus, I do not find support for H5a that the poor should be more responsive to social pressure by neighbors when living in homogeneously poor neighborhoods.
