# Supplementary material for: Neighborly social pressure and collective action: Evidence from a field experiment in Tunisia
Source: PLoS One. 2024 Jul 19;19(7):e0304269. doi: 10.1371/journal.pone.0304269 (PMC11259251; doi:10.1371/journal.pone.0304269)
Supplement: S7 File — (PDF) [file pone.0304269.s020.pdf]

Universität
Konstanz

Universität Konstanz · Box 214 · 78457 Konstanz

Frau
Prisca Jöst-Brenneis, Ph.D.
ExCl 2035

**Sabine Schieß**
**Institutional review board**
**(Ethics Commission)**
Universitätsstraße 10
D-78464 Konstanz
+49 7531 88-5264
Fax +49 7531 88-3727

sabine.schiess@uni-konstanz.de
www.uni-konstanz.de

03.08.2021

# IRB statement 26/2021

Seite: 1/1

To whom it may concern,

in my function as Secretary of the Institutional Review Board (Ethics Committee) of the University of Konstanz I would like to deliver the following statement regarding the research project "Community Participation in Tunisia" which was reviewed at the University of Konstanz as the relevant and most proximate academic institution with an standing institutional review board:

The IRB of the University of Konstanz has evaluated the above proposal, and we have come to the conclusion that this project, and the procedures for data handling and storage are in full line with the ethics regulations of the University of Konstanz.

This judgement was made on following basis:

- the text of the proposal,
- the specific regulations of the University for ethical experimentation and data storage,
- Declaration of Helsinki in its current version as developed by the World Medical Association,
- Relevant national and international law and regulations

The University has an administrative procedure in place that will ensure that ethics guidelines as laid down in German national law and international conventions are fully respected. The IRB will ensure and, if necessary, enforce the proper adherence to ethics guidelines during and after the project.

This IRB approval is given for five years for the specific evaluated project. This also includes additions and prolongations of the project of a technical nature that do not affect the overall judgement of ethical issues and the state of safety of the study subjects.

Sincerely,

Sabine Schieß
