# Supplementary material for: Neighborly social pressure and collective action: Evidence from a field experiment in Tunisia
Source: PLoS One. 2024 Jul 19;19(7):e0304269. doi: 10.1371/journal.pone.0304269 (PMC11259251; doi:10.1371/journal.pone.0304269)
Supplement: S8 File — (PDF) [file pone.0304269.s021.pdf]

Baseline Survey – Tunisia Cleanup Project

**Variable Name**	**Survey Question/ Measures**
WILL BE ANSWERED BY ENUMERATOR:	
Mode of Recruitment	Neighbor vs. stranger
Type of Neighborhood	Homogeneous poor (Le Kram)<br>Mixed poor and wealthy (La Goulette)<br>Homogeneous wealthy (La Marsa)
QUESTIONS FOR PARTICIPANT:	
Density or strength of social ties	How long have you been living in this neighborhood? ENTER MONTHS, YEARS]<br><br><u>Density of social ties:</u><br>In this neighborhood would you say...<br><1> You hardly know anyone at all<br><2> You know few people<br><3> You know many people<br><4> You know almost everyone<br><98> Don't Know/Refuse to answer<br><br>Please think of the neighbors that live on your street. About how many of them do you know by name?<br><1> None or almost none of them<br><2> A few of them<br><3> Many of them<br><4> All of them or almost all of them<br><98> Don't Know/Refuse to answer<br><br><u>Strength of Ties:</u><br>When you think about your neighbors, are most, some, only a few or none of them related to you? (<1> none, <2> only a few, <3> some, <4> most, <98> Don’t know/Refuse to answer)<br><br>When you think about where your friends live, would you say:<br><1> None live in this neighborhood<br><2> Few live in this /neighborhood<br><3> Some, but not most, live in this neighborhood<br><4> Most live in this neighborhood<br><98> Don't Know/Refuse to answer

	How often do you meet others in this neighborhood?<br><1> Almost never<br><2> Rarely<br><3> Frequently<br><4>Very often<br><98> Don't Know/Refuse to answer<br><br>Where do you meet your neighbors?<br><1> At the mosque<br><2> At your workplace<br><3> In the street<br><4> At the market ('fripe')<br><5> At the coffee house<br><7> Other (specify)<br><br>Which are the groups of people you most interact with?<br><1> Religion-based groups<br><2> Classmates<br><3> Colleagues<br><4> Fellow townsmen *(people who are originally from the same region)*<br><5> Political groups<br><6> Other activist groups<br><7> Class-based groups<br><8> Other (specify)<br><br>Which of the following statements best describes your connection with your neighbors?<br><1> We share the same religion.<br><2> We share the same ethnicity.<br><3> We are originally from the same region.<br>*(If not Tunis, please specify <ENTER>)*<br><4> We are colleagues or schoolmates.<br><5> We share the same social class.<br><6> We share the same political views.<br><7> None of these<br><8> Other (specify)
Control Variables	
Demographic Questions	In which of the following age brackets is your age?<br><1> 18-29 <2> 30-39 <3> 40-49 <5> 50-59 <6> 60 + <96> Don't know

	Do you or your household own/have the following:<br>Car/Truck/Van (<1> Yes/ <0> No)<br>Flat screen TV (<1> Yes/ <0> No)<br>Mobile telephone (<1> Yes/ <0> No)<br>Air-conditioner (<1> Yes/ <0> No)<br>Fridge (<1> Yes/ <0> No)<br>Computer (<1> Yes/ <0> No)<br>Swimming pool (<1> Yes/ <0> No)<br><br>Do you feel you are wealthier, the same as, or poorer than most others in this neighborhood?<br>1> Poorer<br><2> The same<br><3> Wealthier<br><97> Don't know<br><98> Refuse to answer<br><br>About how much does your household make in income in a month? (Include all those who make money in this number)<br><ENTER AMOUNT><br><br>I will read out a few statements about your income. Please tell me, which of the following statement is closest to your situation:<br><br><1> Our household income does not cover the needs, there are great difficulties.<br><2> Our household income does not cover the needs, there are difficulties.<br><3> Our household income covers the needs alright, without much difficulty.<br><4> Our household income covers the needs well - we can save.<br><98> Don't Know/Refuse to answer<br><br>Which social class do you belong to?<br><1> Lower class<br><2> Lower-middle class<br><3> Middle class<br><4> Upper-middle class<br><5> Upper class<br><98> Don't Know/Refuse to answer

	Please tell me how many individuals (adults and kids) live in this household (regularly spend the night)?<br>[ENTER NUMBER] Kids<br>[ENTER NUMBER] Adults
Education	What is your highest level of education<br><2> Informal schooling only (including Koranic schooling)<br><3> Some primary schooling<br><4> Primary school completed<br><5> Intermediate school or some secondary school/high school<br><6> Secondary school/high school completed<br><7> Post-secondary qualifications, other than university e.g. a diploma or degree from a polytechnic or college<br><8> Some University<br><9> University completed<br><10> Post-graduate<br><98> Don't know/ Refuse to answer
Employment status	Are you currently employed? (<1> Yes/ <0> No <98> Refuse to answer/Don't know)
Controls	How many free hours do you have in a week (time you do not spend working, caring for kids, sleeping, etc.), including weekends? [ENTER NUMBER OF HOURS]<br><br>On a scale from 0 to 10 where 0 means not at all and 10 very much, how much do you agree with the following statements:<br><br>A trashed environment represents a health risk to me and my family<br><br>I think community clean-up initiatives are a political act<br><br>Keeping public spaces (e.g., the beach, playgrounds, etc.) clean is a civic duty<br><br>How often do you help in volunteer activities or charities either through donating your time or money or other resources? <1> Never, <2> Not often, <3> Sometimes, <4> Very often, <98> Don't know/Refuse to answer<br><br>To what extent do you agree with the following statement: "I am too busy with work or other commitments to

	contribute time to community initiatives." <1> I do not agree, <2> I somewhat agree, <3> I fully agree, <98> Don't know/Refuse to answer<br><br>To what extent do you agree with the following statement: "I would rather contribute money than contribute time to help my community." ." <1> I do not agree, <2> I somewhat agree, <3> I fully agree, <98> Don't know/Refuse to answer
Control: Awareness of Consequences	How much do you think that neighborhood initiatives or events lead to good outcomes for the neighborhood? <1> not much at all, <2> not much, <3> somewhat much, <4> very much, <98> Don't know/Refuse to answer
Control: Expected success of the event	When there are collective activities in this neighborhood, like cleaning a park or helping a family in need, do you think most of your neighbors help, more than half help, less than half help, almost none help? (<1> almost none, <2> less than half, <3> more than half, <4> most, <98> Don't Know/Refuse to answer)<br><br>How many neighbors (*explanation: people who live in your neighborhood but who do not live in the same household)* do you think that you would be able to recruit for a cleanup event if you try? [ENTER NUMBER]
Control: Attribution of Responsibility	What are the reasons for waste accumulation in public spaces around your neighborhood?<br><1> Lack of awareness/education of residents/visitors about how to properly dispose of waste (yes/no)<br><2> Lack of caring of residents/visitors about keeping spaces clean (yes/no)<br><3> Neglect by local political authorities (yes/no)<br><4> Waste collectors pass too infrequent or do not have the right equipment for the<br>job (yes/no)<br><5> Absence of bins into which to throw waste (yes/no)<br><6> Other (specify)<br><br>Can you please tell me to what extent do you hold the following responsible for keeping public spaces clean? (Answer options: (<1> Very much responsible, <2> somewhat responsible, <3>

	not responsible, <4> not at all responsible <98> Don't know/Refuse to answer)<br><br><1> The municipality<br><2> The parliament<br><3> The president<br><4> Private citizens<br><br>Is there someone or some institution other than those mentioned whom you see as responsible for keeping public spaces clean in your area? (<1> Yes/ <0> No)<br><br>If yes, please name the institution <ENTER NAME>
Control: Use of public spaces	Have you come to [*the beach in this neighborhood*] before? (<1> Yes/ <0> No)<br><br>How often have you been to this place in the last six months? <ENTER><br><br>Do you think that you will come to this place in the future? (<1> Yes/ <0> No)
Control: Previous Engagement	Have you participated in any clean-up events in this neighborhood? (<1> Yes/ <0> No)<br><br>Have you participated in any other community events in this neighborhood? (Community meetings, initiatives to feed the poor or tree planting, events at the school, etc.) (<1> Yes/ <0> No)<br><br><u>Political Participation:</u><br>Did you vote in the last municipal elections? (<1> Yes/ <0> No)<br><br>Are you a member of an NGO or other club/organized group? (yes/no)<br>*If so, which one?* <ENTER><br><br>Have you participated in a protest in the past five years? (<1> Yes/ <0> No)

	If the answer is yes, how many times have you participated in protests in the past five years? [ENTER NUMBER OF TIMES]
Other	How much are public spaces like parks, fields or beaches, etc. around your neighborhood in need of cleanup? <1> not at all, <2> not much, <3> somewhat, <4> very much, <98> Don't know/Refuse to answer<br><br>Do you think that [*the beach in this neighborhood*] needs a clean-up? (<1> Yes/ <0> No)

Enumerator Debrief *(will be filled out after the interview by the enumerator)*

Important: ENTER PHONE NUMBER	PHONE NUMBER
Social Status	Based on your impression of the respondent's household, estimate the financial standing of this household in comparison to the other households in this neighborhood:<br><1> Low Income<br><2> Middle Income<br><3> Upper-middle Income<br><4> High Income
Type of Home	What was the type of home?<br><1> An apartment within a building<br><2> Non-detached house (entire building lacking space between its walls and that of another home, but not an apartment within a building)<br><3> Detached house (entire building with space between its walls and other buildings)<br><4> Villa (more than one story high or very large area, property is separated by a wall)
Religious appearance of the respondent or household	How did the respondent/household appear?<br><1> Very religious<br><2> Religious<br><3> Not religious at all
